# Multi‐Omics Profiling of High‐Grade Serous Ovarian Cancer Reveals an Inflammation‐Related Lipid Metabolism Subtype Associated With Platinum Resistance

**DOI:** 10.1002/advs.76482

**Published:** 2026-07-17

**Authors:** Yuxi Zhao, Junyi Li, Bo Zheng, Wanshan Liu, Yaru Wang, Shufeng Wang, Ying Cui, Huiqin Guo, Hongxia Wang, Ting Xiao, Kun Qian, Jing Zuo

**Affiliations:** ^1^ Department of Gynecologic Oncology National Cancer Center/National Clinical Research Center for Cancer/Cancer Hospital Chinese Academy of Medical Sciences and Peking Union Medical College Beijing China; ^2^ State Key Laboratory of Molecular Oncology Department of Etiology and Carcinogenesis National Cancer Center/National Clinical Research Center for Cancer/Cancer Hospital Chinese Academy of Medical Sciences and Peking Union Medical College Beijing China; ^3^ Department of Pathology National Cancer Center/National Clinical Research Center for Cancer/Cancer Hospital Chinese Academy of Medical Sciences and Peking Union Medical College Beijing China; ^4^ State Key Laboratory of Systems Medicine for Cancer School of Biomedical Engineering Institute of Medical Robotics and Shanghai Academy of Experimental Medicine Shanghai Jiao Tong University Shanghai China; ^5^ State Key Laboratory for Oncogenes and Related Genes Shanghai Key Laboratory of Gynecologic Oncology Shanghai China; ^6^ Department of Clinical Trial Research Center Beijing Hospital National Center of Gerontology Institute of Geriatric Medicine Chinese Academy of Medical Sciences Beijing China; ^7^ National Center of Biomedical Analysis Beijing China; ^8^ Institute of Mass Spectrometry Zhejiang Engineering Research Center of Advanced Mass Spectrometry and Clinical Application School of Material Science and Chemical Engineering Ningbo University Ningbo China; ^9^ Zhenhai Institute of Mass Spectrometry Ningbo China

**Keywords:** metabolomics, ovarian cancer, platinum resistance, proteomics, subtyping

## Abstract

Ovarian cancer is the deadliest gynecological malignancy. The main obstacle in treating high‐grade serous ovarian cancer (HGSOC) is platinum resistance. The mechanistic interface between tumor metabolic reprogramming and platinum resistance remains undefined. We integrated proteomic and metabolomic profiling of 97 primary HGSOC tumors to map the molecular landscape. Unsupervised clustering identified three subtypes. Subtype 3 exhibited a higher platinum resistance rate (64.5%), as well as worse overall (*p* = 0.001) and recurrence‐free (*p* = 0.0001) survival. At the molecular level, both Subtypes 2 and 3 tumors displayed phenotypes of activated lipid metabolism. However, Subtype 3 tumors were unique, exhibiting potential divergence between lipid metabolism and bioenergetics. The arachidonic acid metabolism pathway was upregulated (*p* = 2.0 × 10^−6^), and the key enzyme cyclooxygenase‐2 (COX‐2) was overexpressed (*p* < 0.001). Subtype 3 tumors exhibited an inflammation‐associated state with the highest infiltration of M2‐like macrophages (*p* = 0.007). Single‐cell transcriptomics revealed increased expression of *PTGS2* (encoding COX‐2, *p* = 0.001) and *CD163* (*p* < 2.2 × 10^−16^) in macrophages from platinum‐resistant HGSOC. Multiplex immunofluorescence confirmed that platinum‐resistant tumors had a higher proportion of COX‐2^+^ cells in M2‐like macrophages (*p* = 0.010). These findings define a high‐risk HGSOC subtype characterized by inflammation‐related lipid metabolism. This suggests targeting the arachidonic acid metabolism pathway as a way to overcome platinum resistance.

## Graphical abstract

Through integrated proteomic and metabolomic profiling, Zhao et al. identified three molecular subtypes of high‐grade serous ovarian cancer. The high‐risk subtype exhibits activated arachidonic acid metabolism and cyclooxygenase‐2 overexpression. This metabolic axis promotes M2‐like macrophage infiltration, which contributes to platinum resistance. These findings identify potential therapeutic targets for overcoming chemoresistance.

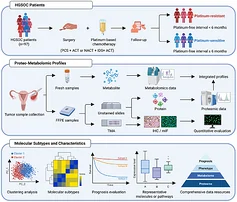

## 1 Introduction

Ovarian cancer (OC) remains the most lethal gynecological malignancy [1]. High‐grade serous OC (HGSOC) accounts for approximately 70% of cases, with the majority of patients diagnosed at advanced stages [2]. Standard first‐line treatment for OC consists of primary cytoreductive surgery followed by platinum‐based adjuvant chemotherapy, and most patients achieve a clinical response. Nevertheless, disease recurrence occurs in over 70% of cases within a three‐year period [3]. Although management for recurrent OC relies primarily on platinum‐based chemotherapy, cumulative exposure leads to progressive acquisition of platinum resistance. This phenotype represents the primary obstacle to improving the poor 5‐year overall survival (OS) rate of 31% [4]. Elucidating the molecular mechanisms underlying platinum resistance is critical to developing effective therapeutic strategies.

In the past decades, large‐scale molecular profiling efforts, such as The Cancer Genome Atlas (TCGA) [5] and the Clinical Proteomic Tumor Analysis Consortium (CPTAC) [6], have significantly advanced our understanding of HGSOC heterogeneity by delineating genomic, transcriptomic, and proteomic subtypes. For instance, the TCGA classification consists of immunoreactive, differentiated, proliferative, and mesenchymal subtypes, which have important implications for patient outcomes. Furthermore, a recent study [7] has identified four immune categories among treatment‐naïve ovarian tumors that were also associated with prognosis. However, genomic landscapes prove insufficient in revealing the real‐time functional dynamics within tumor tissues, particularly the rapid adaptations in protein stability and metabolic flux that occur under therapeutic stress. Consequently, a robust molecular subtyping strategy that directly links proteomics and metabolomics to platinum resistance is still lacking.

Proteomics and metabolomics were selected for this study due to their close proximity to the functional phenotype. Although genomics and transcriptomics provide insights into genetic alterations and transcriptional potential, these measurements do not always correlate with the actual functional state of the cell [8]. Proteomics identifies the protein effectors of cellular signaling, while metabolomics characterizes the biochemical activities and metabolic endpoints. Chemoresistance of HGSOC can be driven by post‐translational adaptations [9] and metabolic reprogramming [10, 11]. Metabolic fingerprinting has shown promise for OC diagnosis [12]. Integrating these two omics layers facilitates the identification of therapeutic targets and provides insight into the heterogeneity of the disease [13].

Resistance to platinum is a multifactorial phenomenon. Recent advances highlight metabolic reprogramming as a critical mechanism by which HGSOC escapes from chemotherapy‐induced cell death [14]. HGSOC exhibits marked adipotropism, preferentially metastasizing to lipid‐rich omental tissue [15]. In this niche, tumor‐derived extracellular vesicles remodel both stromal adipocytes and macrophages, triggering lipolysis and lipid droplet accumulation in tumor‐associated macrophages (TAMs). This metabolic cross‐talk drives TAMs toward an immunosuppressive M2‐like phenotype, which in turn confers chemoresistance via pro‐survival cytokine release and extracellular matrix remodeling [16, 17]. Despite these insights, the specific lipid metabolic pathways that interface with the tumor immune microenvironment to drive platinum resistance remain undefined [18], underscoring the need to investigate the immunometabolism synapse where metabolic reprogramming and immune evasion converge [19].

To bridge these gaps, we applied an integrated proteomic‐metabolomic approach to 97 primary HGSOC tumors, aiming to identify metabolic subtypes associated with platinum resistance. Here, we identify a high‐risk, platinum‐resistant molecular subtype characterized by a profound activation of arachidonic acid metabolism. We demonstrate that this specific lipid‐inflammatory axis fosters an M2‐like macrophage‐enriched microenvironment, thereby providing a mechanistic framework and potential targets for platinum resistance in HGSOC.

## 2 Materials and Methods

### 2.1 Patient Enrollment, Treatment, Sample Collection, and Follow‐Up

The study was approved by the Ethics Committee of the Cancer Hospital of the Chinese Academy of Medical Sciences (No. 23/221‐3936) and was in accordance with the principles of the Declaration of Helsinki. The requirement for written informed consent was waived given the retrospective design. Samples and clinical data were collected from 97 patients between April 2010 and May 2019, including 93 with pure HGSOC and 4 with mixed components.

Patients either underwent primary cytoreductive surgery (PCS, *n* = 58) or received neoadjuvant chemotherapy (NACT) for two to three cycles followed by interval debulking surgery (IDS, *n* = 39). The choice of treatment modality was determined by the possibility of complete resection, guided by CT and/or MRI scanning: if complete resection with no visible residual tumor was deemed feasible, patients underwent PCS; otherwise, they received NACT followed by IDS. All patients received platinum‐based chemotherapy after surgery. The standard chemotherapy regimen was paclitaxel 175 mg/m^2^ and carboplatin AUC 4‐5.

Intraoperatively collected tumor tissue samples were processed immediately. A portion was formalin‐fixed and paraffin‐embedded (FFPE) for proteomics analysis, and the remaining tissue was stored at −80°C for subsequent metabolomics analysis.

The platinum sensitivity was classified based on the platinum‐free interval (PFI), calculated from the date of the last administration of platinum‐based chemotherapy to the date of the next recurrence. Patients with a PFI ≥ 6 months were classified as platinum‐sensitive, while those with a PFI < 6 months were classified as platinum‐resistant.

### 2.2 Proteomics Analysis

Mass spectrometry for proteomics analysis was performed on FFPE samples (*n* = 89) using a Thermo Q Exactive HF tandem mass spectrometry (MS/MS) system via Data Independent Acquisition (DIA). Protein identification was performed with a 1% FDR threshold, requiring a minimum of two peptides per protein and allowing two missed cleavages. Quantification was conducted via a DIA approach using Spectronaut (Biognosys, Schlieren, Switzerland) based on the extracted ion chromatograms (XICs) of precursor and fragment ions [20]. Proteins expressed in at least half of the patients were included for downstream analysis, and missing protein values were imputed using the Bayesian principal component analysis (BPCA) method.

### 2.3 Metabolomic Analysis

Using an ultra‐high performance liquid chromatography‐tandem electrospray ionization mass spectrometry (UHPLC‐ESI‐MS/MS) approach, fresh samples (*n* = 94) were analyzed by both reversed‐phase and hydrophilic interaction liquid chromatography (RPLC and HILIC). Quality control samples were pooled from the subject samples and inserted into each set of runs to monitor stability. Non‐targeted metabolomics utilized a TripleTOF 6600 QTOF‐MS coupled to a Shimadzu LC‐30AD UHPLC system with TurboV ESI interface, operating in dual ionization modes (ESI+/−) per chromatographic analysis. The technical details of the metabolomics analysis were described in our previous publication [21]. The raw data were processed using the Progenesis QI software (Waters, Milford, MA) according to established workflows [22, 23]. Peak detection was performed using the co‐detection algorithm in Progenesis QI. This generated an aggregate ion map across all samples. Peak picking used sensitivity‐based thresholding (the default setting) and a minimum peak width of 3. We conducted alignment using a reference‐run‐based vector approach to correct for retention time shifts. The default processing pipeline was used to apply baseline correction and smoothing. Then, normalization was applied using the total ion count (TIC) method. Missing metabolomics values were imputed using K‐nearest neighbors (KNN) method. Metabolites with variable importance in projection (VIP) values for platinum resistance greater than 1.0 were included in the molecular subtyping process.

### 2.4 Molecular Subtype Classification

The CancerSubtypes package [24] was used to integrate proteomics and metabolomics data for molecular subtyping of patients. We performed integrative clustering using the ExecuteiCluster() function. Input matrices were log2‐transformed and standardized prior to analysis. For patients with incomplete omics data, we computed Euclidean distances between each such sample and all patients in the fully profiled cohort. Each incomplete sample was then assigned to the subtype of its nearest neighbor based on minimal Euclidean distance in the available omics space. Data visualization of the multi‐omics profiles was performed using the ComplexHeatmap R package. Prior to clustering, rows (proteins and metabolites) were standardized by *Z*‐score transformation. Hierarchical clustering of both features and samples was conducted based on the Euclidean distance using the complete linkage algorithm.

### 2.5 Differential Analysis and Pathway Enrichment

The Limma package [25] and Wilcoxon rank‐sum test were used to examine whether proteins, metabolites, and pathways were differentially expressed between different subtypes. The metabolic and immune pathways were characterized using gene set variation analysis (GSVA) [26] based on the Kyoto Encyclopedia of Genes and Genomes (KEGG) database [27, 28, 29].

### 2.6 Single‐Cell Transcriptomic Analysis

Single‐cell transcriptomics data [30] of a total of 45 144 cells from the primary tumors of 10 HGSOC patients were obtained from Mendeley Data. To maintain consistency across datasets, the 10 patients in the public single‐cell cohort were categorized using the same PFI‐based criteria (≥ 6 months for sensitivity and < 6 months for resistance) as applied to our primary cohort. The Seurat v4.4.0 pipeline was used for data preprocessing, and the top 30 principal components were used for uniform manifold approximation and projection (UMAP) and nearest‐neighbor graph construction. Annotation of major cell populations aligned with the insights provided by the original study. Cell subpopulations were clustered using the Louvain algorithm and were manually annotated based on their highly expressed genes. The SCP package v0.5.6 was used for visualization.

### 2.7 Immunohistochemical/Immunofluorescence Staining

The expression of key candidate proteins in tissues from different molecular subtypes was validated by immunohistochemical staining on the BenchMark GX system (Roche, Switzerland). The antibodies used included anti‐VIM (Cat No. 5741, Cell Signaling, USA), anti‐CD163 (Cat No. 68218‐1‐Ig, Proteintech, USA), and anti‐COX‐2 (Cat No. MA5‐14568, Invitrogen, USA) antibodies. Two independent pathologists evaluated the IHC slides while being blinded to the clinical outcomes. The expression of COX‐2 and vimentin (VIM) was quantified using the H‐score, which ranges from 0 to 300. The infiltration level of CD163+ macrophages was determined by calculating the percentage of positive cells out of the total number of cells. Furthermore, tissue microarrays were created using FFPE tissue samples from 25 HGSOC patients (platinum‐resistant, *n* = 9; platinum‐sensitive, *n* = 16). Multiplex immunofluorescence (mIF) staining was performed according to standard operating procedure of Cell DIVE (Leica Microsystems, Germany). After dewaxing and hydration, two‐step antigen retrieval was performed. Nuclear staining was performed according to the Cell DIVE DAPI staining protocol, and the background autofluorescence was recorded. The antibodies used included anti‐COX‐2 (Cat No. MA5‐14568, Invitrogen, USA), anti‐CD68 (Cat No. sc‐20060, Santa Cruz, USA), anti‐CD163 (Cat No. nb110‐40686ir, NOVUS, USA), and anti‐PanCK (Cat No. 53‐9003‐82, Invitrogen, USA) antibodies. Cell DIVE output results were analyzed using the HALO digital pathology image analysis platform to determine the expression of each marker and the negative and positive cutoff values of each marker for each cell. The PanCK^+^ cells were defined as tumor cells. The CD68^+^CD163^+^ cells were defined as M2‐like macrophages. Then, the proportion of each cell type that was COX‐2^+^ was calculated for each case.

### 2.8 Statistical Analysis

Statistical analyses were performed using R (v4.4.1) and MedCalc (v19.6.1). Associations between categorical variables were assessed using Fisher's exact test. The Kruskal–Wallis *H* test was used to assess significant differences across three groups, and the Wilcoxon rank sum (Mann–Whitney *U*) test was used for pairwise comparisons. We estimated survival probabilities using the Kaplan–Meier method and compared them using the two‐sided log‐rank test. The optimal cutoff value for stratifying patients into high‐ and low‐activity groups based on arachidonic acid pathway activity was 0.29, as determined by receiver operating characteristic (ROC) curve analysis using MedCalc software (version 19.6.1) with survival status as the endpoint. All statistical tests were two‐sided, with an alpha level set at 0.05. Multiple testing correction was performed using the Benjamini–Hochberg false discovery rate (FDR) method for the differential analyses of global proteomic and metabolomic expression profiling, as well as comprehensive pathway enrichment analyses.

## 3 Results

### 3.1 Clinical Characteristics and Prognosis of HGSOC Patients

The clinical characteristics of the patients are shown in Figure 1A and Table S1. Platinum‐resistant patients had significantly worse prognoses than platinum‐sensitive patients in terms of OS (24.3 vs. 89.9 months, *p* < 0.0001) and recurrence‐free survival (RFS) (3.0 vs. 16.5 months, *p* < 0.0001) (Figure 1B, Figure S1A,B).

**FIGURE 1:**
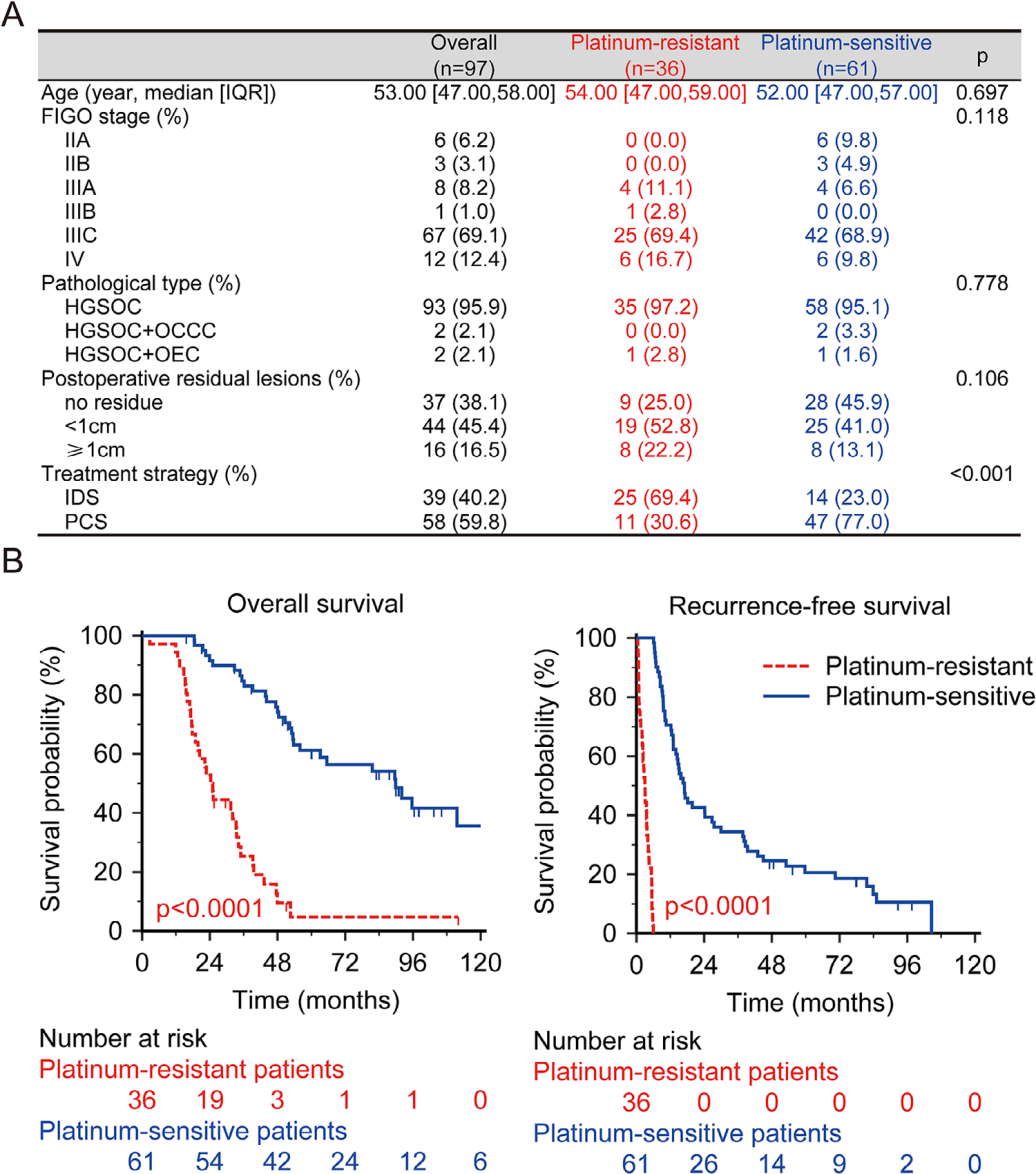
Clinical information and prognosis of platinum‐sensitive (*n* = 61) and platinum‐resistant (*n* = 36) ovarian cancer patients. (A) Clinical characteristics. (B) Overall survival (left panel) and recurrence‐free survival (right panel) outcomes. Associations between categorical variables were analyzed using Fisher's exact test. IQR, interquartile range. FIGO, International Federation of Gynecology and Obstetrics. HGSOC, high‐grade serous ovarian carcinoma. OCCC, ovarian clear cell carcinoma. OEC, ovarian endometrioid carcinoma. IDS, interval debulking surgery. PCS, primary cytoreductive surgery.

### 3.2 Proteomic and Metabolomic Profiling of HGSOC Patients

In the proteomic analysis, we identified a total of 8708 proteins, with an average of 7146 proteins identified per sample. All samples passed the quality control with an expected unimodal distribution. Protein quantification metrics, including total numbers and abundance levels, remained comparable across all four conditions (*p* > 0.05, Figure S2A,B). After quality control of the proteomics data, 7450 proteins were included for downstream analysis. The expression distribution of the proteomics data was relatively uniform, indicating that the quality control of the proteomics data was well executed. Through principal component analysis of the proteomic data (Figure S2C), we found that proteomics alone could not distinguish between platinum‐resistant and platinum‐sensitive patients.

In the metabolomics analysis, a total of 10 151 metabolic features were detected by combining RPLC‐ESI‐MS and HILIC‐ESI‐MS. A total of 427 metabolites with platinum sensitivity‐derived VIP > 1 were obtained and annotated for downstream analysis. The metabolite numbers and abundance showed no significant differences among four conditions (*p* > 0.05, Figure S2D,E). Metabolomics data alone were also insufficient to distinguish between platinum‐resistant and platinum‐sensitive patients (Figure S2F).

### 3.3 Proteo‐Metabolomic Molecular Subtyping Predicts Platinum Resistance and Patient Survival

To explore the sources of intertumoral heterogeneity in HGSOC and its relationship with platinum‐sensitivity, we integrated proteomic and metabolomic data to perform molecular subtyping through iCluster algorithm using CancerSubtypes package [24]. Based on a comprehensive consideration of the silhouette coefficient and clinical significance, the patients were classified into three subtypes, which showed a significant correlation between molecular subtype and platinum resistance (*p* = 0.0007) (Figure 2A, Figure S3A,B). The platinum resistance rate was notably higher in S3 (64.5%) compared to S1 (25.0%) and S2 (23.7%) patients. Prognostic analysis of these three subtypes revealed that S3 patients had inferior OS (*p* = 0.0010) and RFS (*p* = 0.0001) compared to S1 and S2 patients (Figure 2B).

**FIGURE 2:**
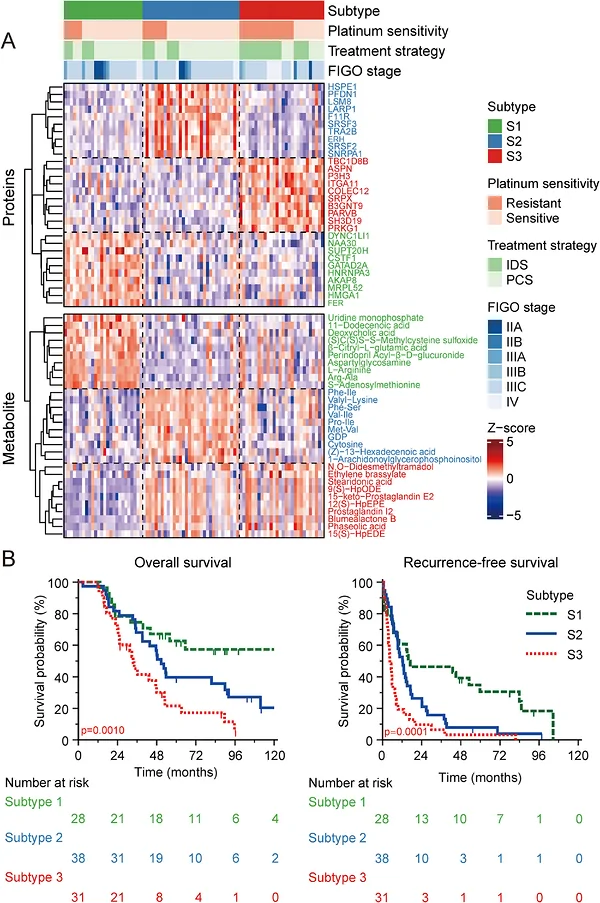
Molecular subtypes of ovarian cancer patients (*n* = 97) based on integrated proteo‐metabolomic analysis. (A) Representative highly expressed proteins or high‐abundance metabolites of ovarian cancer patients with different molecular subtypes. The color blocks in the top bar reflect the clinical conditions of the patient. Protein and metabolite expression levels were standardized using *Z*‐score transformation across rows to ensure comparability, with the color scale representing relative abundance ranging from −5 (*blue*) to 5 (*red*). Hierarchical clustering was performed using the Euclidean distance metric and the complete linkage method. (B) Differences in overall survival (left panel) and recurrence‐free survival (right panel) among patients with different molecular subtypes. FIGO, International Federation of Gynecology and Obstetrics. IDS, interval debulking surgery. PCS, primary cytoreductive surgery.

### 3.4 Subtype‐Specific Metabolic Pathway Activation

We characterized major metabolic categories across the three subtypes based on KEGG pathways (Figure 3A,B, Figure S3C). S1 tumors exhibited the lowest levels of lipid metabolism (*p* = 1.6 × 10^−5^) and xenobiotics biodegradation and metabolism (*p* = 6.1 × 10^−5^). In contrast, both S2 and S3 tumors displayed elevated lipid metabolism, yet diverged in their functional utilization of lipid substrates. S2 tumors coupled high lipid metabolism with elevated energy metabolism (*p* = 0.0087), suggesting active fatty acid oxidation for bioenergetic support. In S3 tumors, a potential divergence between lipid metabolism and bioenergetic pathways was observed. These tumors exhibited suppressed energy metabolism while maintaining high lipid metabolic activity. This suggests that lipid substrates may be channeled toward non‐energetic processes instead of mitochondrial oxidation. Furthermore, S3 tumors exhibited the lowest levels of nucleotide metabolism (*p* = 0.0013), consistent with a metabolic state prioritizing stress adaptation over rapid proliferation.

**FIGURE 3:**
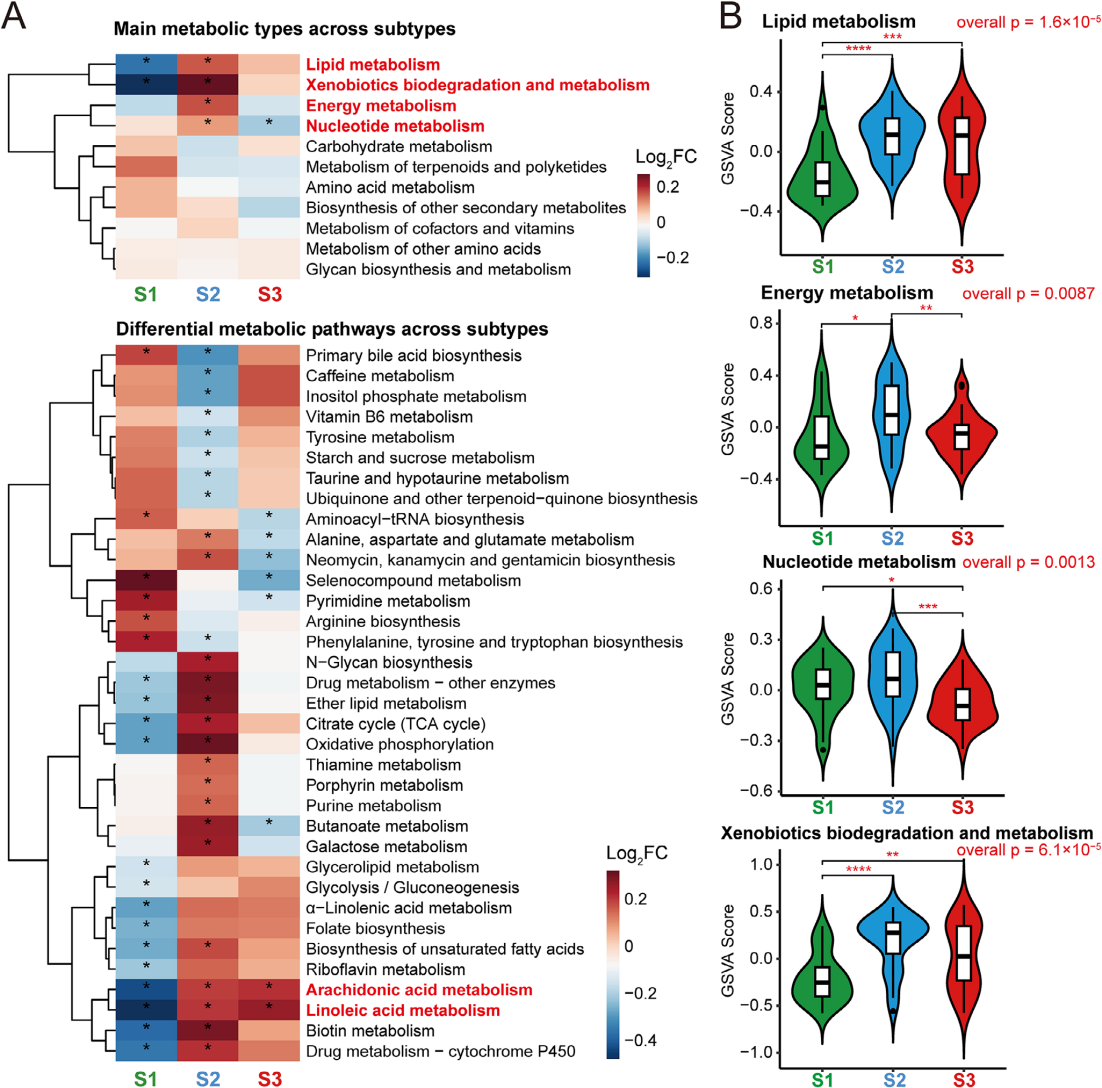
Metabolic characteristics in different molecular subtypes of ovarian cancer patients (*n* = 97). (A) Activation status of major metabolic types (top) and metabolic pathways (bottom) across different molecular subtypes. (B) Significant differences in metabolic pathways including lipid metabolism, energy metabolism, nucleotide metabolism, and xenobiotics biodegradation and metabolism among the three subtypes. FC, fold change. The Kruskal–Wallis *H* test was used for comparisons among three subtypes, with the Mann–Whitney *U* test for subsequent pairwise comparisons. *p < 0.05. **p < 0.01. ***p < 0.001. ****p < 0.0001.

### 3.5 Association Between Arachidonic Acid Pathway Activation and Patient Prognosis

Analysis of the specific lipid pathways driving the S2 and S3 phenotypes revealed a convergence on inflammatory lipid precursors. Both S2 and S3 patients exhibited activated lipid biosynthesis and metabolism phenotype, including significant upregulations of the arachidonic acid metabolism (*p* = 2.0 × 10^−6^), the linoleic acid metabolism (*p* = 4.3 × 10^−6^) and the biosynthesis of unsaturated fatty acids (*p* = 0.0051) (Figure 4A). Among these, increased flux of linoleic acid metabolism can elevate the level of arachidonic acid. Importantly, high arachidonic acid metabolism was significantly associated with reduced OS (*p* = 0.0001) and RFS (*p* = 0.0005), suggesting that this pathway may serve as a driver of aggressive disease (Figure 4B). In addition, there were certain differences in other metabolic pathways among the three subtypes (Figures S3D and S4).

**FIGURE 4:**
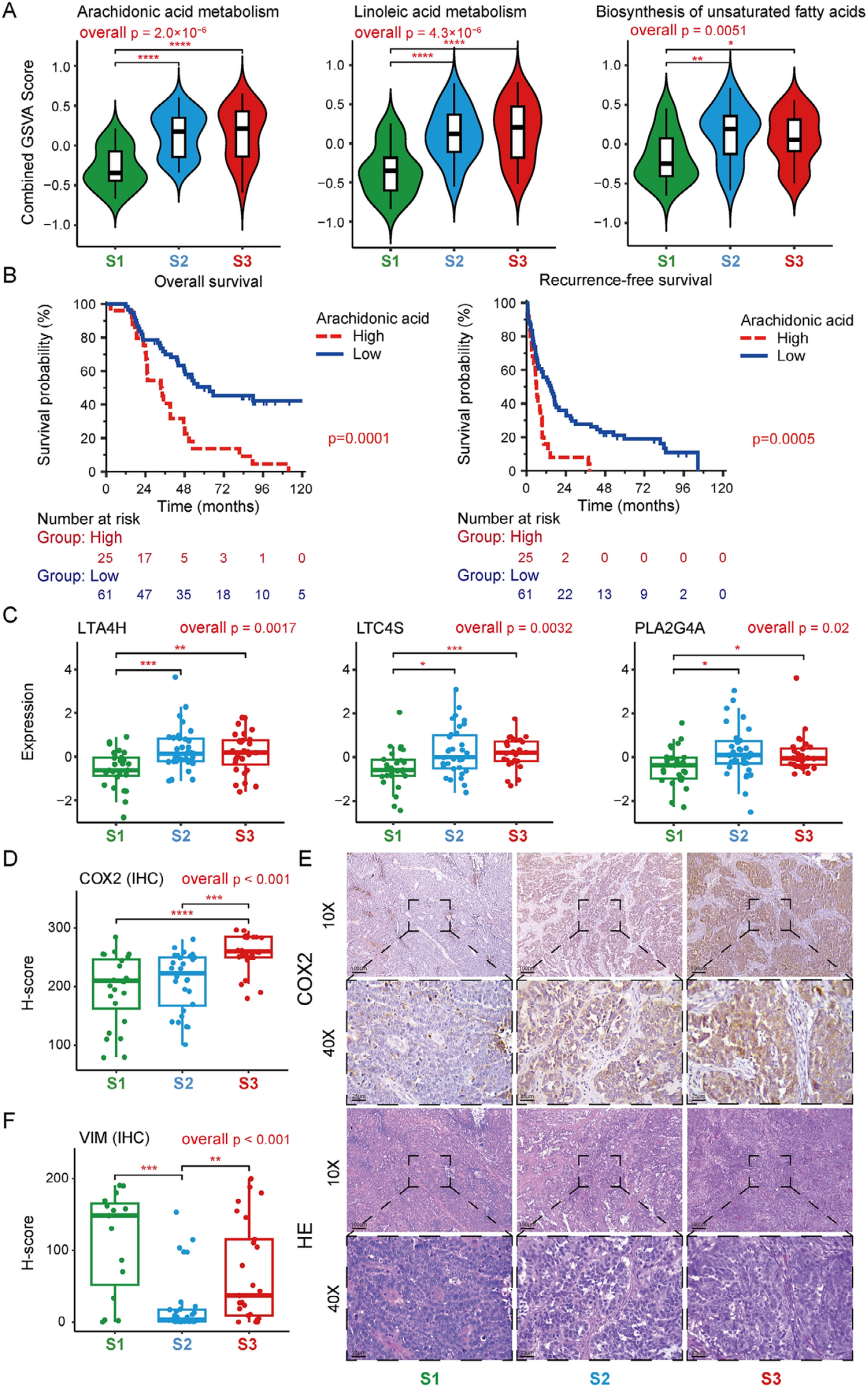
Correlation of arachidonic acid pathway activation with patients' (*n* = 97) molecular subtypes and prognosis. (A) Activation status of lipid metabolism‐related pathways in different subtypes. (B) Relationship between the level of arachidonic acid pathway activation and patient's overall survival (left panel) and recurrence‐free survival (right panel). (C) Expression levels of key proteins in the arachidonic acid metabolism pathway: LTA4H (left panel), LTC4S (middle panel), and PLA2G4A (right panel). (D) COX‐2 expression levels in patients with the three subtypes assessed by immunohistochemistry. (E) Representative staining images for immunohistochemical assessment of COX‐2 expression levels. (F) VIM expression levels in patients with the three subtypes assessed by immunohistochemistry. IHC, immunohistochemistry. HE, hematoxylin‐eosin staining. The Kruskal–Wallis *H* test was used for comparisons among three subtypes, with the Mann–Whitney *U* test for subsequent pairwise comparisons. *p < 0.05. **p < 0.01. ***p < 0.001. ****p < 0.0001. Scale bars: 100 µm (10×) and 25 µm (40×).

To identify the key enzymes accounting for this metabolic shift, we used the proteomics data to explore the key mediators of the arachidonate cascade. We found that key proteins in arachidonic acid metabolism pathways, such as LTA4H (Kruskal–Wallis *H* test, *p* = 0.0017), LTC4S (*p* = 0.0032), and PLA2G4A (*p* = 0.02), were significantly upregulated in both S2 and S3 patients (Figure 4C, Figure S5). When stimulated by inflammation or inflammatory mediators, arachidonic acid is metabolized through cyclooxygenase (COX) and lipoxygenase (LOX) pathways, generating various metabolites, with prostaglandins and leukotrienes being the most important. Among them, COX‐1 is mainly involved in physiological functions (such as gastric mucosal protection and platelet aggregation), while COX‐2 is mainly increased in expression under pathological conditions such as inflammation, infection, and cancer. Immunohistochemical staining revealed a critical divergence between the two lipid‐rich subtypes regarding COX‐2. We observed that COX‐2 expression levels in S3 patients were significantly higher (*p* < 0.001) than those in S1 and S2 patients (Figure 4D,E). In addition, the expression of VIM—a protein significantly downregulated in S2 patients—was also validated via immunohistochemistry (Figure 4F, Figure S6). Collectively, these data define S3 as a unique COX‐2‐high arachidonic acid metabolism subtype with a poor prognosis.

### 3.6 S3 Patients Exhibited an Inflammatory Phenotype With Elevated Macrophage Infiltration

Considering the important role of metabolic‐immune crosstalk in tumor progression, we further characterized the immune microenvironment across the three subtypes. Based on proteomic data, we found that S3 patients exhibited a more active inflammatory state, while S2 patients demonstrated lower levels of inflammation (Figure 5A, Figure S7A). We observed the highest levels of macrophage infiltration (*p* = 0.007) and chemokine receptor (*p* = 0.012) expression in S3 patients, yet their HLA expression levels showed no significant elevation (*p* = 0.18), nor did they exhibit markedly enhanced overall cytolytic activity (*p* = 0.52) (Figure 5B). This suggests that the infiltration of macrophages is predominantly M2‐like for S3 patients. Furthermore, we also observed a discrepancy between the inflammatory level and the activation level of antigen‐presenting cells. Although this inflammatory state may be related to interferon and is mediated jointly by neutrophils, macrophages, T cells and B cells, the immune checkpoint expressions increased (Figure S7B). Using immunohistochemistry, we confirmed elevated infiltration of M2‐like macrophages in the tumor stroma of both S2 and S3 patients, which was statistically significant for the S3 subtype (Figure 5C,D).

**FIGURE 5:**
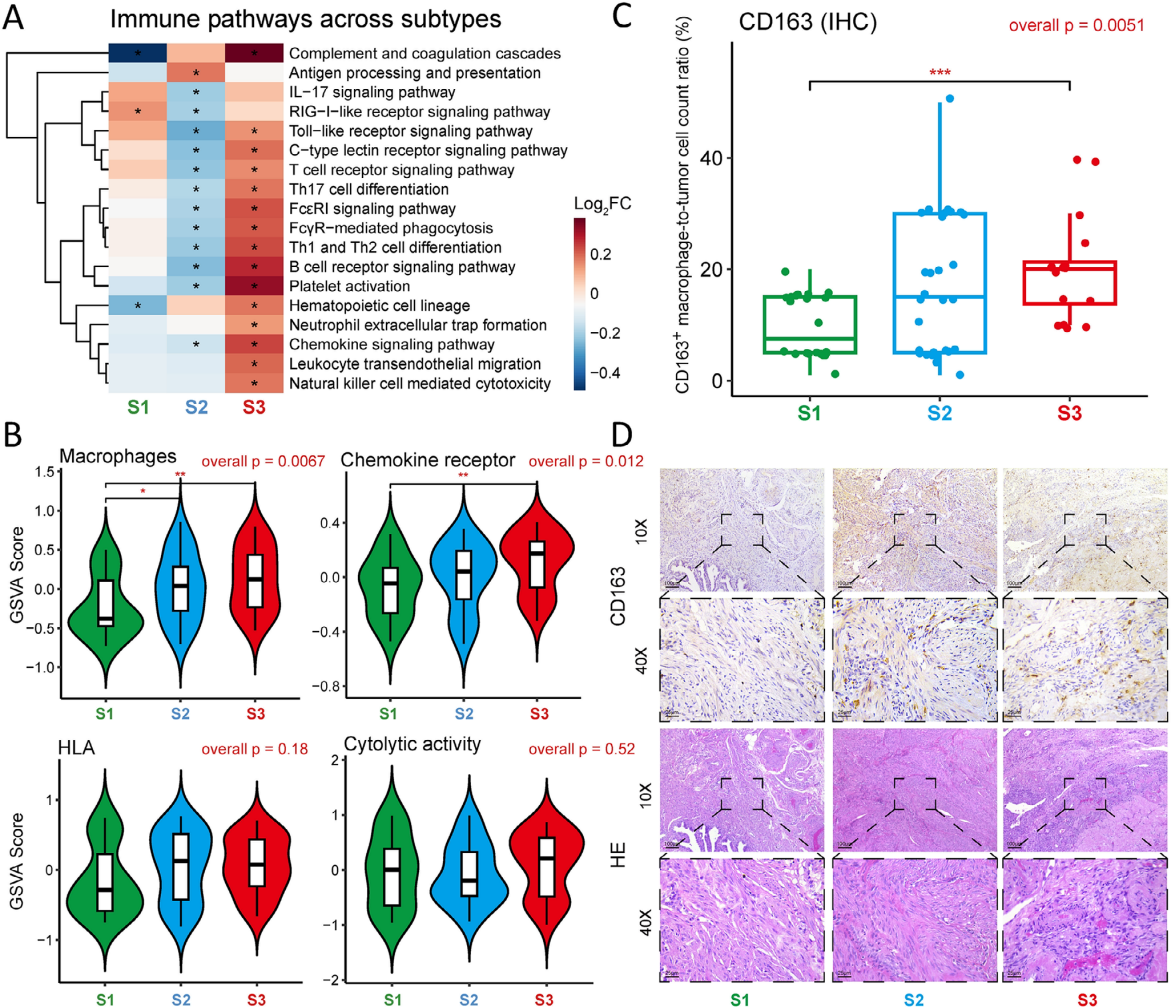
Immune characteristics of ovarian cancer patients (*n* = 97) with different molecular subtypes. (A) Immune pathway activation across different molecular subtypes. (B) Abundance of molecules related to macrophages, chemokine receptors/human leukocyte antigen, and cytotoxic activity in different subtypes. HLA, human leukocyte antigen. (C) CD163 expression levels in patients with the three subtypes assessed by immunohistochemistry. (D) Representative staining images for immunohistochemical assessment of CD163 expression levels. HE, hematoxylin‐eosin staining. The Kruskal–Wallis *H* test was used for comparisons among three subtypes, with the Mann–Whitney *U* test for subsequent pairwise comparisons. *p < 0.05. **p < 0.01. ***p < 0.001. Scale bars: 100 µm (10×) and 25 µm (40×).

### 3.7 Single‐Cell Transcriptome Analysis Revealed Platinum‐Resistant Patients had Higher Levels of PTGS2 and CD163 Expression in Macrophages

To further validate the association between macrophage infiltration and platinum resistance, we analyzed single‐cell transcriptomics data of 10 HGSOC patients from a previous study [30] (Figure S8A). We observed higher levels of macrophage infiltration in tumors from platinum‐resistant patients (Figure 6A,B). *PTGS2*, the gene encoding COX‐2, was predominantly expressed in macrophages (Figure 6C). Macrophages were clustered in an unsupervised manner into nine clusters based on the Louvain algorithm (Figure 6D, Figure S8B). *S100A8* ^+^ proinflammatory macrophages (*p* = 1.27 × 10^−112^), *RPS10* ^+^ translation‐activated macrophages (*p* = 5.11 × 10^−16^) and *EGR1* ^+^ early‐activated/stressed macrophages (*p* = 4.11 × 10^−21^) exhibited higher *PTGS2* expression levels (Figure 6E). Macrophages in the resistant group had higher expression of *PTGS2* (p = 0.0013) and *CD163* (p < 2.2×10^−16^) (Figure 6F,G). These findings corroborate the link between COX‐2‐expressing M2‐like macrophages and platinum resistance observed in our bulk proteomic analysis.

**FIGURE 6:**
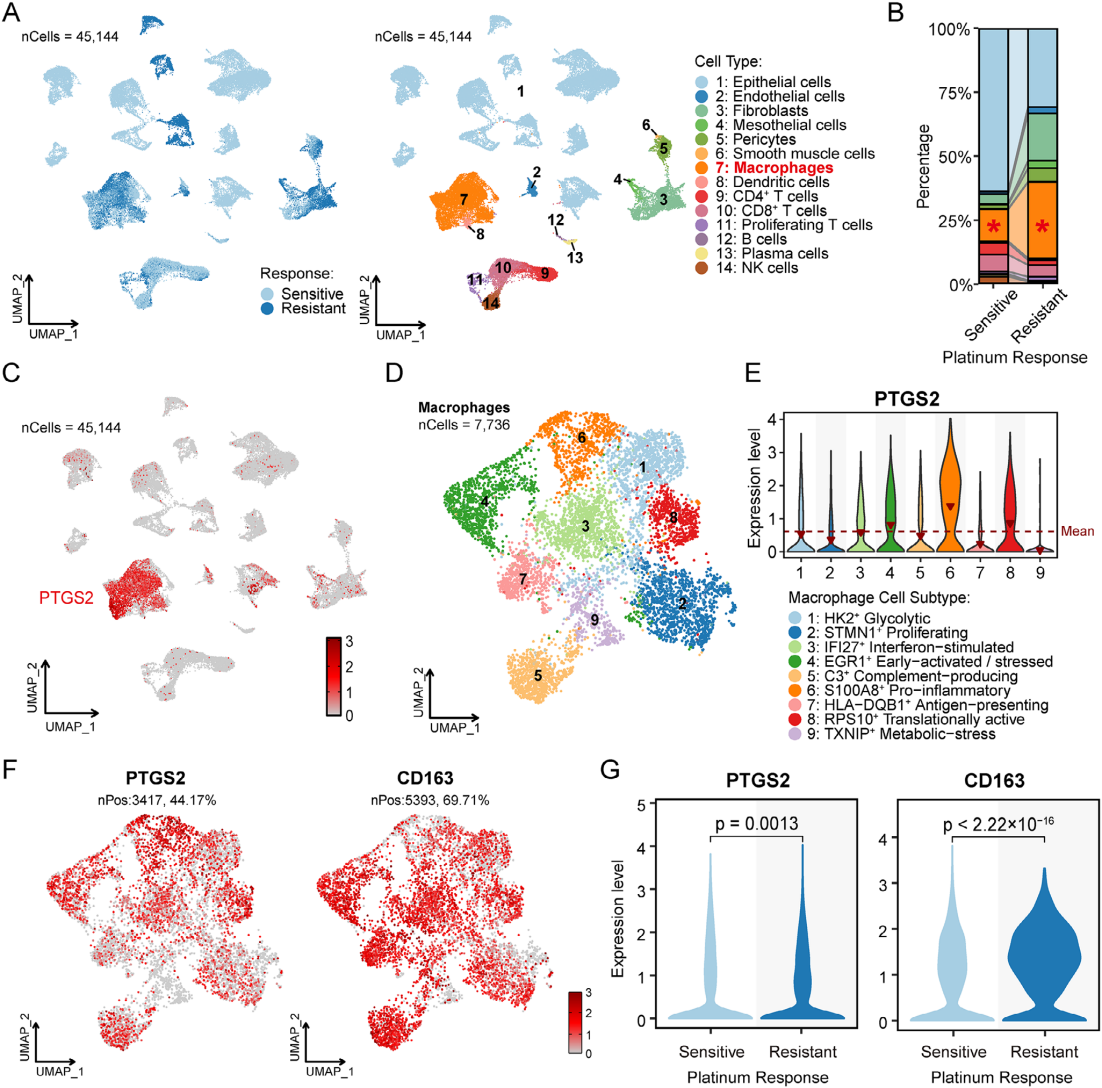
Single‐cell transcriptomic analysis reveals elevated *PTGS2* and *CD163* expressions in macrophages of platinum‐resistant HGSOC (*n* = 10). (A) A total of 45 144 cells from 10 HGSOC primary tumors, colored by platinum response status (left panel) and major cell type annotations (right panel). (B) The proportion of major cell types in platinum‐sensitive and platinum‐resistant tumors. (C) The expression of *PTGS2* across all cell types, highlighting its predominant expression in macrophages. (D) Macrophages were clustered using unsupervised clustering into nine distinct subpopulations. (E) The expression level of *PTGS2* across the nine identified macrophage subtypes. (F) The co‐expression patterns of *PTGS2* and *CD163* within the macrophage population. (G) The expression levels of *PTGS2* (left panel) and *CD163* (right panel) in macrophages between platinum‐sensitive and platinum‐resistant patient groups. Statistical significance was determined by the Mann–Whitney *U* test. UMAP, uniform manifold approximation and projection.

### 3.8 Elevated COX‐2 is Associated With Platinum Resistance of OC

To spatially validate COX‐2 expression in the tumor microenvironment and its association with platinum resistance, we performed mIF staining. COX‐2 expression was observed in tumor cells as well as in M2‐like macrophages within the tumor microenvironment (Figure 7A). Quantification revealed a significantly higher proportion of COX‐2^+^ cells in macrophages (20.84% [95% CI: 10.63%–54.88%] vs. 4.25% [95% CI: 2.82%–22.41%], *p* = 0.003) and M2‐like macrophages (25.53% [95% CI: 12.90%–53.30%] vs. 3.77% [95% CI: 2.58%–15.80%], *p* = 0.010) in the resistant group compared to the sensitive group (Figure 7B,C). These results establish COX‐2 as a marker of platinum resistance and suggest that targeting the COX‐2/arachidonic acid axis may represent a promising therapeutic strategy for S3 patients.

**FIGURE 7:**
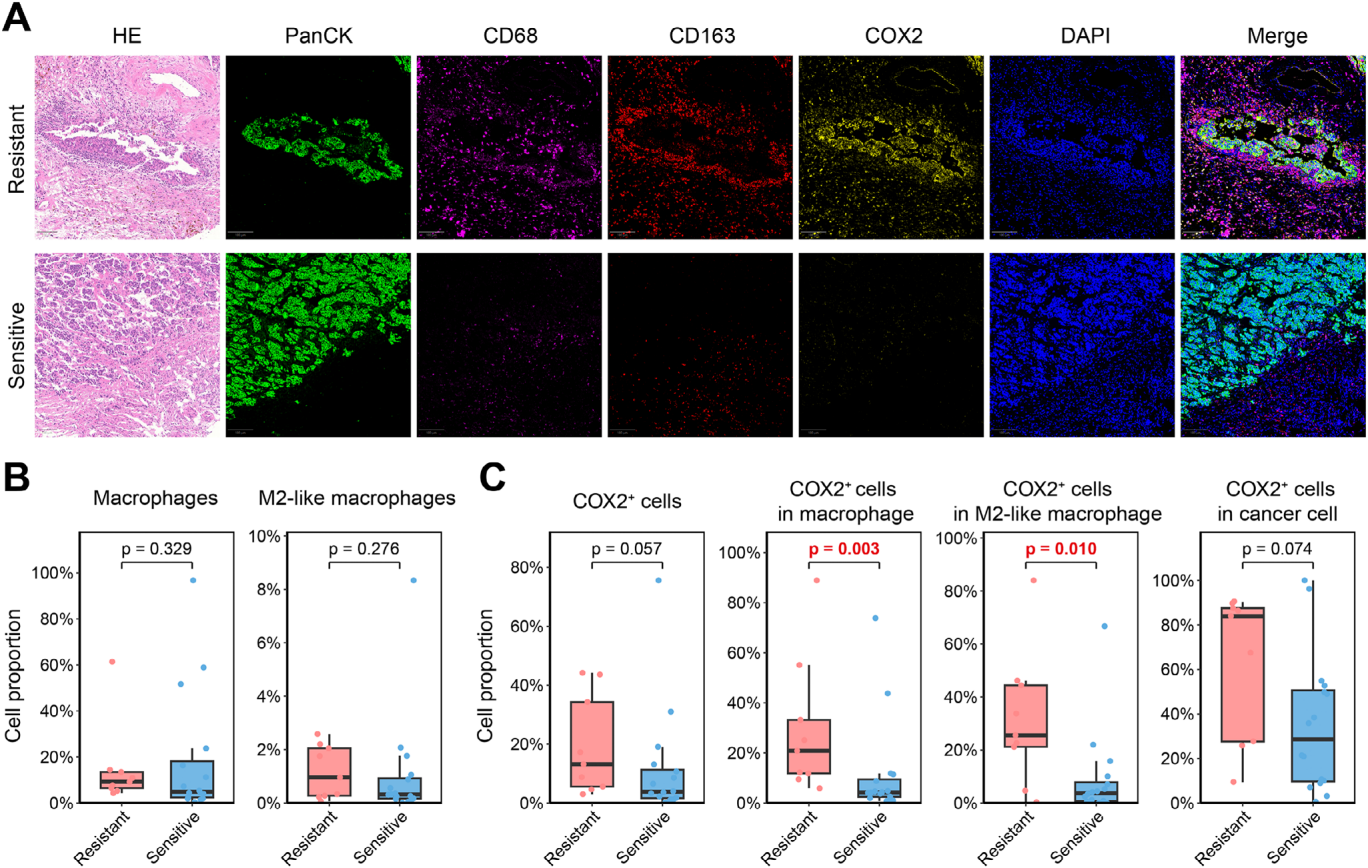
Characterization of COX‐2 expression in tumor cells and macrophages via multiplex immunofluorescence. (A) Representative patients from the platinum‐resistant or platinum‐sensitive group. (B) Proportions of total macrophages and M2‐like macrophages relative to total nucleated cells in the resistant (*n *= 9) and sensitive (*n* = 16) groups. (C) Comparison of the proportions of COX‐2+ cells within total nucleated cells, macrophages, M2‐like macrophages, and cancer cells between the resistant (*n* = 9) and sensitive (*n* = 16) groups. Scale bars: 100 µm. Statistical significance was determined by the Mann–Whitney *U* test.

## 4 Discussion

In this comprehensive multi‐omics investigation, we utilized proteomic and metabolomic profiling to establish three distinct molecular subtypes (S1, S2, and S3) of HGSOC with distinct metabolic profiles, microenvironmental characteristics and platinum response. Our findings reveal that the lipid‐metabolism‐dominant subtype (S3) is characterized by elevated arachidonic acid metabolism, COX‐2 overexpression, and pronounced M2‐like macrophage infiltration. These results indicate that a coordinated immunometabolism relationship exists between arachidonic acid metabolism and the platinum resistance frequently observed in S3 patients, with potential implications for both prognosis and therapeutic strategies.

### 4.1 Integrating Multi‐Omics to Refine Subtyping in HGSOC

Despite advances in genomics and transcriptomics, molecular subtyping of HGSOC has remained inconsistent across studies. Genomic events such as *TP53* mutations [31], *BRCA1/2* mutations [32], *CCNE1* amplification [33], and copy number alterations [34] have been recognized as drivers of chemoresistance. However, these events do not fully account for platinum‐resistant phenotypes. In addition, discrepancies across transcriptional studies [35, 36] and challenges in reproducing subtype labels across cohorts [37] have limited their clinical utility. Our study, by combining proteomics and metabolomics, provides a function‐based classification that reflects tumor characteristics at the protein and metabolic levels. Such integration has the potential to overcome the limitations of genomic or transcriptomic classifications, which often capture upstream regulatory information rather than real‐time biological behavior [38].

This study employed proteomics and metabolomics on FFPE and fresh tissues, respectively. FFPE tissue is an established resource for clinical proteomics. Using FFPE tissue allows for the concurrent processing of all samples in a single batch, which minimizes inter‐sample batch effects. Previous studies have confirmed that FFPE preservation maintains sufficient proteomic integrity for clinical subtyping [39, 40], showing a high correlation with fresh‐frozen samples (Pearson's *r*: 0.93–0.97) [41]. Metabolomics was used on fresh tissue to preserve transient biochemical states and prevent the degradation of reactive metabolites during formalin fixation. To ensure the reliability of the data integration process, we used a matched‐sample design. For each patient, both tissue types were collected simultaneously from the same surgical site, ensuring biological synchronization between the two molecular layers.

Using proteomics and metabolomics data, we identified three metabolically distinct HGSOC subtypes. Patients in S1 were characterized by the lowest levels of lipid metabolism and xenobiotic biodegradation. In contrast, both S2 and S3 tumors exhibited relatively high levels of lipid metabolism. Although there was no increase in carbohydrate metabolism, the S2 subtype exhibited elevated energy metabolism. This pattern indicates a metabolic shift toward fatty acid oxidation to sustain cellular energetics. This is consistent with the concept of metabolic reprogramming in cancer, whereby tumor cells adapt to stress by relying on alternative energy sources [42]. These shifts may provide S2 tumors with the metabolic intermediates and ATP necessary to survive chemotherapy‐induced stress. However, the platinum‐resistant S3 tumors exhibited a unique metabolic paradox. Despite having high lipid metabolic activity, they displayed suppressed energy metabolism. This discrepancy suggests that lipid substrates in S3 tumors may be redirected toward non‐energetic processes, such as the production of signaling mediators.

Both our S1 and S3 subtypes exhibit high VIM expression, which overlaps with the TCGA [5] “Mesenchymal” and the CPTAC [6] “Stromal‐high” subtypes. However, our integrated analysis reveals an unrecognized layer of metabolic heterogeneity between them. The S1 subtype is characterized by low lipid metabolic activity. In contrast, the S3 subtype exhibits activation of lipid metabolism, particularly arachidonic acid metabolism. This is coupled with platinum resistance and high infiltration of M2‐like macrophages. Therefore, our findings suggest that the mesenchymal‐like subtype in HGSOC is not homogeneous. It may be driven by distinct metabolic‐immune axes. By identifying the specific lipid and inflammatory features within the S3 subtype, our study may reveal new metabolic targets that could help overcome chemoresistance in this most aggressive subtype.

### 4.2 Arachidonic Acid Metabolism and Platinum Resistance

S3 tumors exhibited significant enrichment of lipid metabolism, particularly arachidonic acid and linoleic acid pathways. Arachidonic acid has long been implicated in tumor progression through its conversion into bioactive lipids such as prostaglandins and leukotrienes [43]. Our data reveal that metabolites of this pathway, including prostaglandin I2, 15‐keto‐prostaglandin E2, 20‐hydroxy‐leukotriene B4, 5‐HETE and 12‐HETE, were significantly elevated in S3 tumors, with increased expression of COX‐2, LTA4H, LTC4S, and PLA2G4A. These findings strongly implicate the arachidonic acid‐COX‐2‐prostaglandin axis in platinum resistance and poor prognosis, consistent with prior work linking arachidonic acid metabolites to angiogenesis, proliferation, and immune evasion [44].

Importantly, our findings highlight that elevated COX‐2 expression was observed in both tumor cells and CD163^+^ M2‐like macrophages, implicating the arachidonic acid‐COX‐2‐prostaglandin axis as a central immunometabolism driver of resistance. Previous studies demonstrated that COX‐2 positivity was significantly more frequent in non‐responders to platinum‐based chemotherapy, suggesting COX‐2 as a predictor of chemoresistance in OC [45]. Furthermore, a recent review highlighted the pivotal role of arachidonic acid metabolism, including COX pathways, in OC progression and as a potential target for therapy [46]. In alignment with this evidence, our study provides further evidence by linking the arachidonic acid metabolism pathway to platinum resistance via an immune‐metabolic interface. Future research based on single‐cell metabolic analysis [47] may further explore this observation.

### 4.3 Immunometabolism Relationship With M2‐Like Macrophages Reinforces Platinum Resistance

The immunologic characteristic of the S3 subtype is defined by macrophage enrichment. COX‐2‐mediated arachidonic acid metabolism may induce functional changes in multiple components of the tumor microenvironment [48]. TAMs, particularly M2‐like CD163^+^ subsets, are recognized facilitators of chemoresistance through secretion of pro‐survival cytokines, extracellular matrix remodeling enzymes, and angiogenic mediators [49]. By examining the spatial distribution of COX‐2 and CD163, we observed that COX‐2 expression extends from tumor cells to infiltrating macrophages. Arachidonic acid metabolites may promote the polarization of macrophages toward an M2‐like state. It is hypothesized that COX‐2+ TAMs sustain prostaglandin‐driven immunosuppression. This proposed feedback regulation is associated with a permissive microenvironment that may result in platinum resistance. However, further functional studies are needed to confirm these causal links.

Beyond macrophages, elevated arachidonic acid impairs NK cell function via disruption of key cytotoxic signaling pathways, such as STAT1 and activating receptors, compromising immune surveillance and contributing to poor progression‐free survival [48]. This further expands the immunological impact of arachidonic acid beyond macrophages to broader immune dysfunction, supporting the significance of this metabolic axis in tumor resistance and progression.

### 4.4 Strengths and Limitations of This Study

The strengths of this study include the integration of proteomic and metabolomic datasets, validating them using immunohistochemistry and mIF, and cross‐confirming key findings using publicly available single‐cell transcriptomics data. Our findings regarding the key role of COX‐2 in HGSOC align with recent functional studies [7]. A streamlined prognostic panel integrating our identified COX‐2 IHC scoring and key lipid metabolism enzymes with established clinical indicators such as serological CA125 and HE4, *BRCA1/2* and homologous recombination deficiency status, and PD‐L1 and Ki‐67 expression could offer a more accessible route for patient stratification. Besides, mass spectrometry‐based metabolic fingerprinting has demonstrated emerging applications in clinical diagnostics [50, 51, 52]. Following further clinical research and large‐scale validation, this integrated approach may enable rapid identification of patients' subtypes, guiding personalized combinations of metabolic inhibitors with immunotherapy, PARP inhibitors, or conventional chemotherapy.

However, certain limitations exist. First, while our cohort of HGSOC patients is moderate in size for an integrated proteo‐metabolomic study, the generalizability of the identified molecular subtypes still requires further confirmation. The conclusions of this study would be strengthened by independent external validation. Our multicentric external validation is in progress. Next, in‐depth functional experiments, such as evaluating the potential of COX‐2 inhibition to restore platinum sensitivity, will be crucial. Second, due to the long storage period required for the prognostic correlation analysis, antigen loss was observed in some FFPE specimens. This reduced the final mIF set available for statistical analysis to 25 cases. Consequently, these results only serve as supportive validation and must be confirmed in larger studies. Third, the causal relationship between COX‐2‐positive M2‐like macrophages and the tumor immune microenvironment requires future mechanistic elucidation. Notably, targeted therapies against arachidonic acid metabolism may offer a novel therapeutic strategy for S3 patients. In particular, drugs that inhibit key enzymes of the COX and LOX pathways (e.g., COX‐2 inhibitors) may reverse platinum resistance. This provides an opportunity to develop individualized treatment strategies for patients with HGSOC.

## 5 Conclusion

In conclusion, this study identified clinically relevant molecular subtypes of OC using integrated proteomic and metabolomic analysis. Specifically, we defined a platinum‐resistant molecular subtype in HGSOC, characterized by a poor prognosis, a lipid‐metabolism‐dominant phenotype and elevated macrophage infiltration. We found that the activation of the arachidonic acid metabolism pathway and the infiltration of COX‐2‐positive M2‐like macrophages are associated with platinum resistance. The immunometabolism relationship may imply a potential biomarker‐informed treatment strategy, aimed at overcoming platinum resistance and improving outcomes of platinum‐resistant OC.

## Author Contributions

**Yuxi Zhao**: resources, investigation, writing original draft, methodology, validation. **Junyi Li**: investigation, formal analysis, visualization, writing original draft. **Bo Zheng**: resources, investigation, writing original draft, validation. **Wanshan Liu**: validation, writing review and editing, investigation, methodology. **Yaru Wang**: data curation, investigation, formal analysis, writing original draft. **Shufeng Wang**: data curation, formal analysis, writing review and editing. **Ying Cui**: validation, writing review and editing. **Huiqin Guo**: validation, writing review and editing, resources. **Hongxia Wang**: resources, methodology, writing review and editing, funding acquisition, supervision. **Ting Xiao**: resources, supervision, writing review and editing, funding acquisition, methodology. **Kun Qian**: conceptualization, resources, writing review and editing, methodology, funding acquisition, supervision. **Jing Zuo**: conceptualization, resources, writing review and editing, funding acquisition, supervision.

## Funding

This study was supported by the National Key Research and Development Program of China (No. 2022YFF0705004), the National Natural Science Foundation of China (Nos. 82503438, 82273120, 92557304, and W2511090), the Chinese Academy of Medical Sciences Innovation Fund for Medical Sciences (Nos. 2023‐I2M‐2‐004, and 2022‐I2M‐C&T‐B‐082), and the Yongjiang Talent Project of Ningbo Science and Technology Innovation (No. 2023A‐148‐G).

## Conflicts of Interest

The authors declare no conflicts of interest.

## Supporting information




**Supporting File 1**: advs76482‐sup‐0001‐SuppMat.pdf.

**Supporting File 2**: advs76482‐sup‐0002‐TableS1.xlsx.

## Data Availability

The data that support the findings of this study are available from the corresponding author upon reasonable request. The raw proteomic and metabolic data have been deposited in the OMIX database (accession number OMIX017023 and OMIX008340). The raw microscope image data of multiplex immunofluorescence and hematoxylin‐eosin staining for the tissue microarray were uploaded to Zenodo (https://doi.org/10.5281/zenodo.20131882). The de‐identified clinical characteristics of patients are listed in Table S1.
