# Supplementary material for: Multi‐Omics Profiling of High‐Grade Serous Ovarian Cancer Reveals an Inflammation‐Related Lipid Metabolism Subtype Associated With Platinum Resistance
Source: Adv Sci (Weinh). 2026 Jul 17;13(55):e76482. doi: 10.1002/advs.76482 (PMC13377746; doi:10.1002/advs.76482)
Supplement: Supplementary file 1 [file ADVS-13-e76482-s002.pdf]

Supporting Information for

Multi-Omics Profiling of High-Grade Serous Ovarian Cancer Reveals an Inflammation-Related Lipid Metabolism Subtype Associated with Platinum Resistance

Yuxi Zhao[#], Junyi Li[#], Bo Zheng[#], Wanshan Liu[#], Yaru Wang, Shufeng Wang, Ying Cui, Huiqin Guo, Hongxia Wang[*], Ting Xiao[*], Kun Qian[*], Jing Zuo[*]

Table of contents

Supplementary figures (1-8)

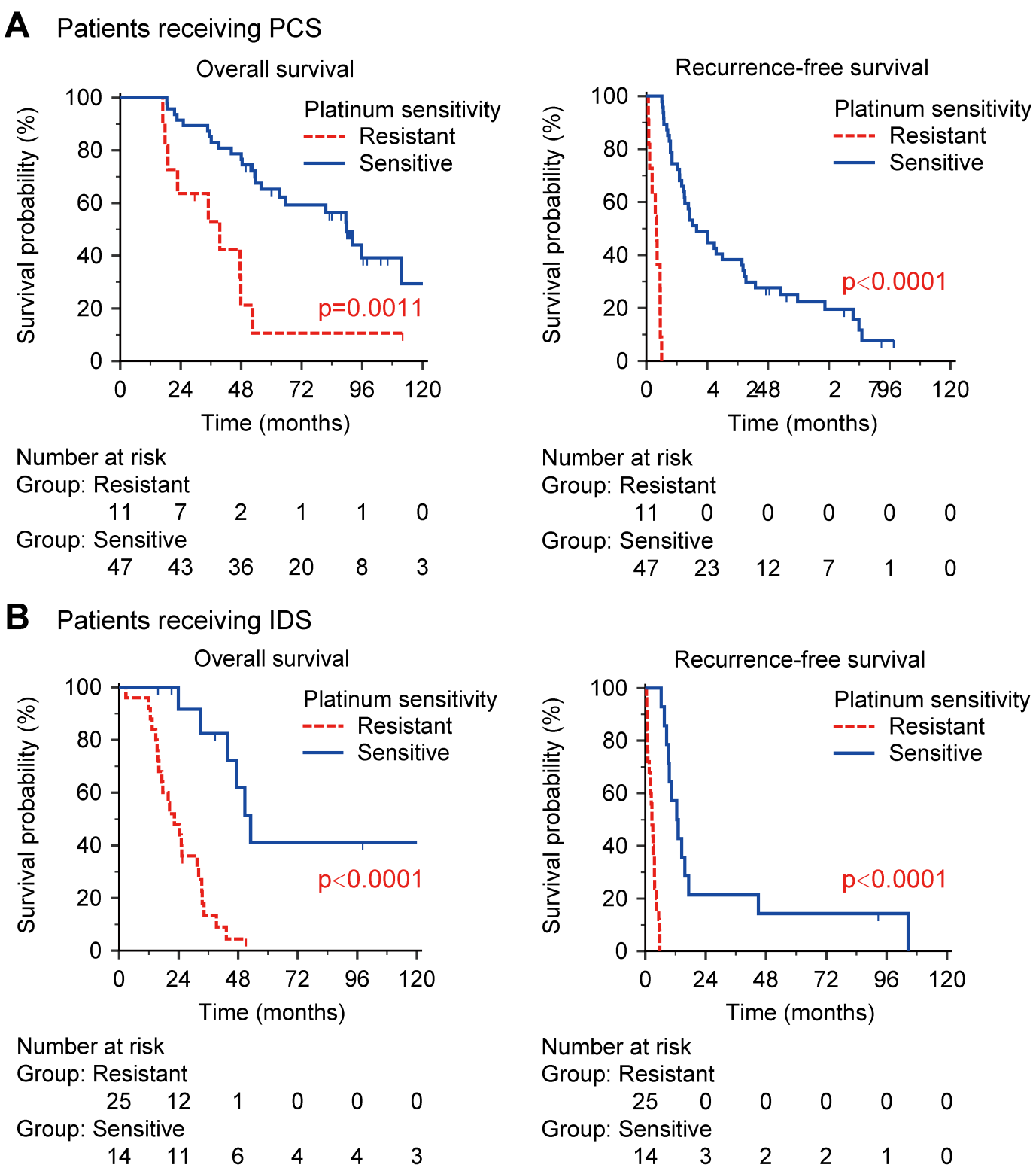

Fig. S1. Patient prognosis based on primary cytoreductive surgery (PCS) or interval debulking surgery (IDS). (Supplement to Figure 1.) **(A)** Overall survival (OS, left) and recurrence-free survival (RFS, right) for patients who underwent PCS. **(B)** OS (left) and RFS (right) for patients who underwent IDS. PCS, primary cytoreductive surgery; IDS, interval debulking surgery.

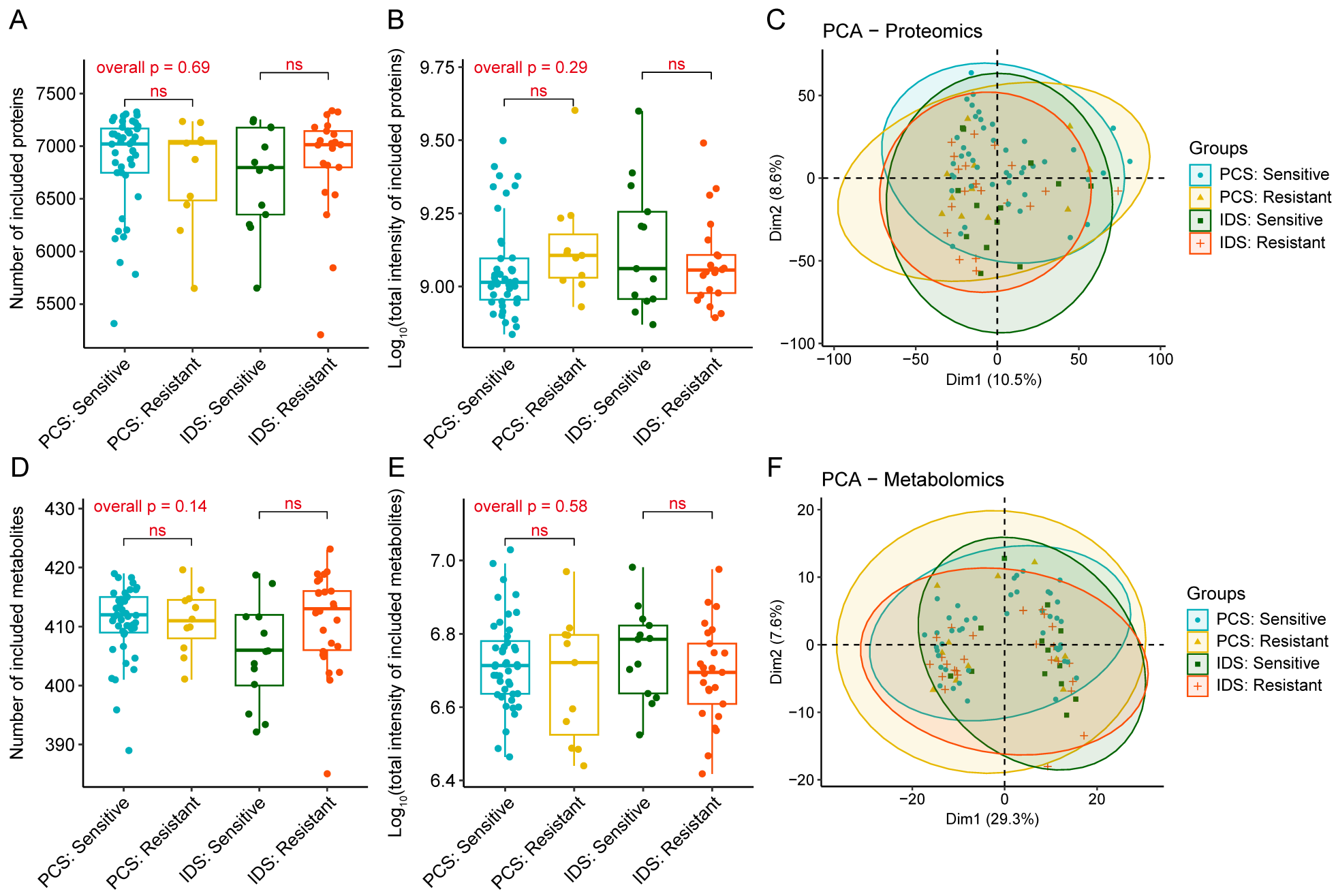

Fig. S2. Overview of proteomic and metabolomic data across different treatment strategies and platinum sensitivities. (A) Number of proteins included in the analysis. **(B)** Total intensity of included proteins. **(C)** Distribution of proteomic data classified by group, based on principal component analysis. **(D)** Number of metabolites included in the analysis. **(E)** Total intensity of included metabolites. **(F)** Distribution of metabolomics data classified by group, based on principal component analysis. PCS, primary cytoreductive surgery; IDS, interval debulking surgery. PCA, principal component analysis.

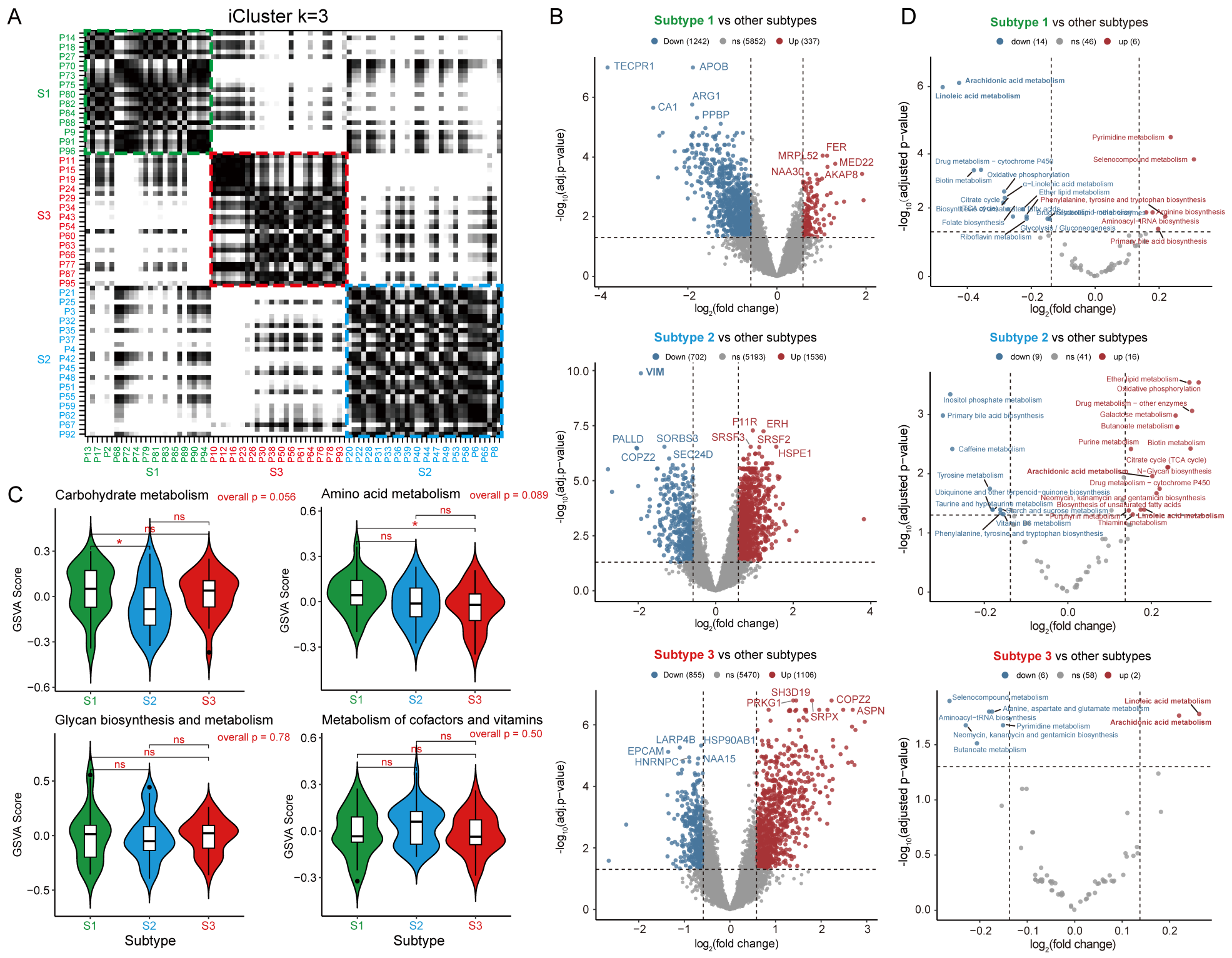

Fig. S3. Metabolic characteristics in different molecular subtypes of ovarian cancer patients. (A) Correlation heatmap showing patient clustering based on the iCluster algorithm (Supplement to Figure 2A). **(B)** Proteins significantly differentially expressed among patients with the three subtypes (adjusted $p < 0.05$, fold change > 1.5) (Supplement to Figure 2A). **(C)** Activation status of major metabolic types across different molecular subtypes (Supplement to Figure 3). **(D)** Metabolic pathway differences between each subtype and the remaining subtypes (Supplement to Figure 3). The Kruskal-Wallis H test was used for comparisons among three subtypes, with the Mann-Whitney U test for subsequent pairwise comparisons. ns, no significant difference. * $p < 0.05$.

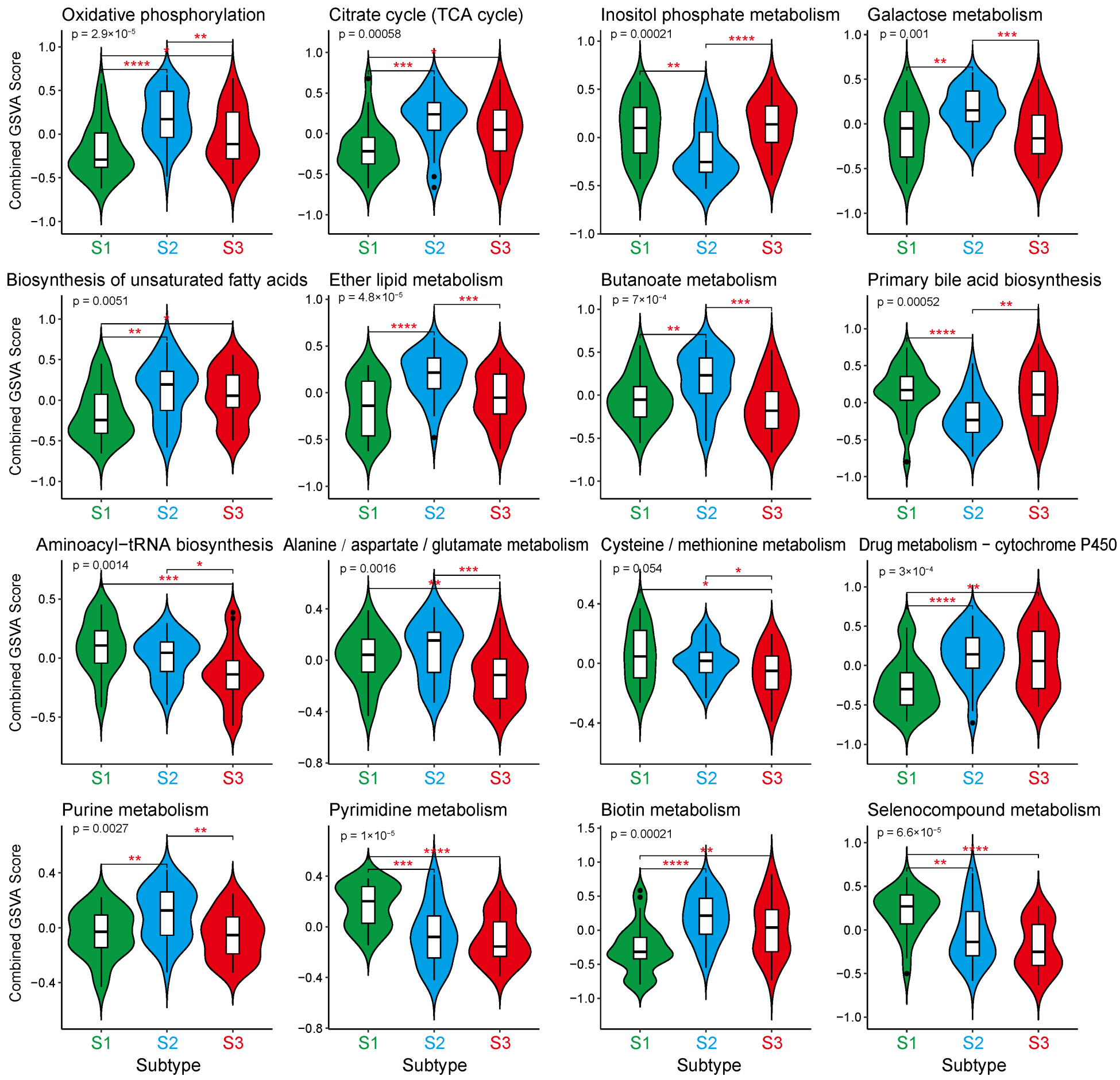

Fig. S4. Abundance of selected metabolic pathways in different molecular subtypes. (Supplement to Figure 3.) The Kruskal-Wallis H test was used for comparisons among three subtypes, with the Mann-Whitney U test for subsequent pairwise comparisons. * $p < 0.05$. ** $p < 0.01$. *** $p < 0.001$. **** $p < 0.0001$.

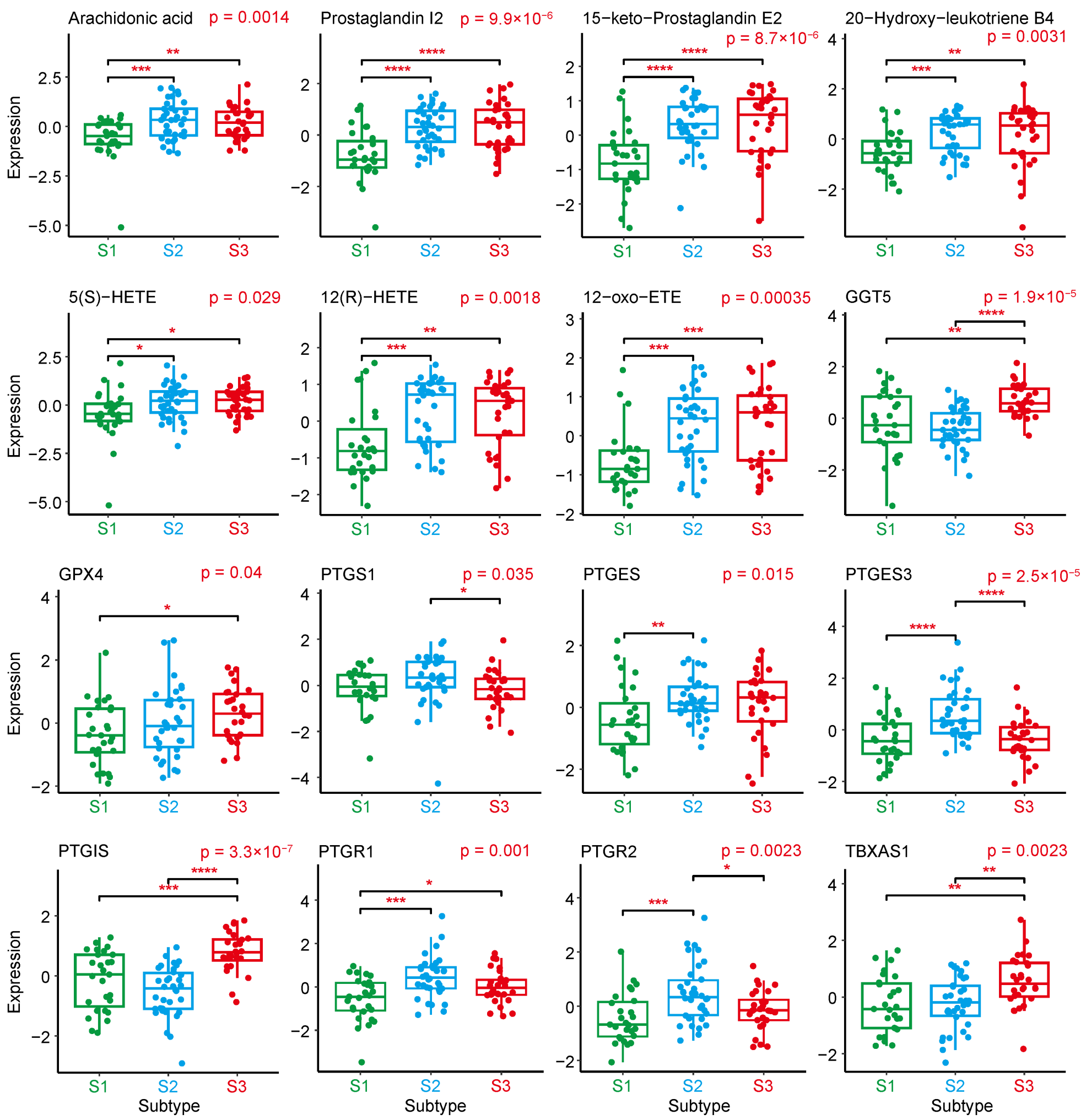

Fig. S5. Expression or abundance of arachidonic acid metabolism-related metabolites and key proteins in different subtypes. (Supplement to Figure 4C.) The Kruskal-Wallis H test was used for comparisons among three subtypes, with the Mann-Whitney U test for subsequent pairwise comparisons. * $p < 0.05$. ** $p < 0.01$. *** $p < 0.001$. **** $p < 0.0001$.

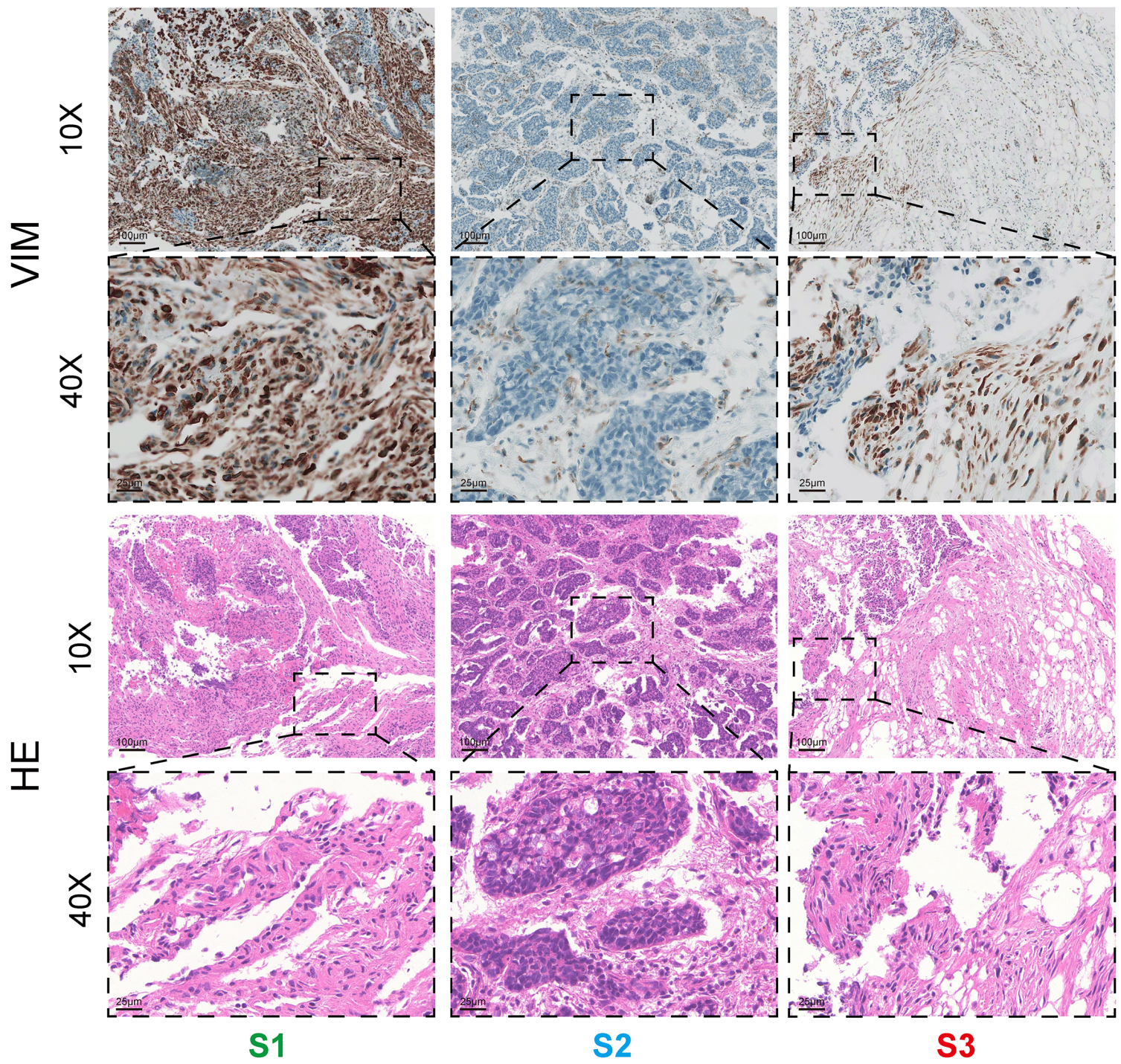

Fig. S6. Representative staining images for immunohistochemical assessment of VIM expression levels. (Supplement to Figure 4F.) HE, hematoxylin-eosin staining. Scale bars: 100 μm (10×) and 25 μm (40×).

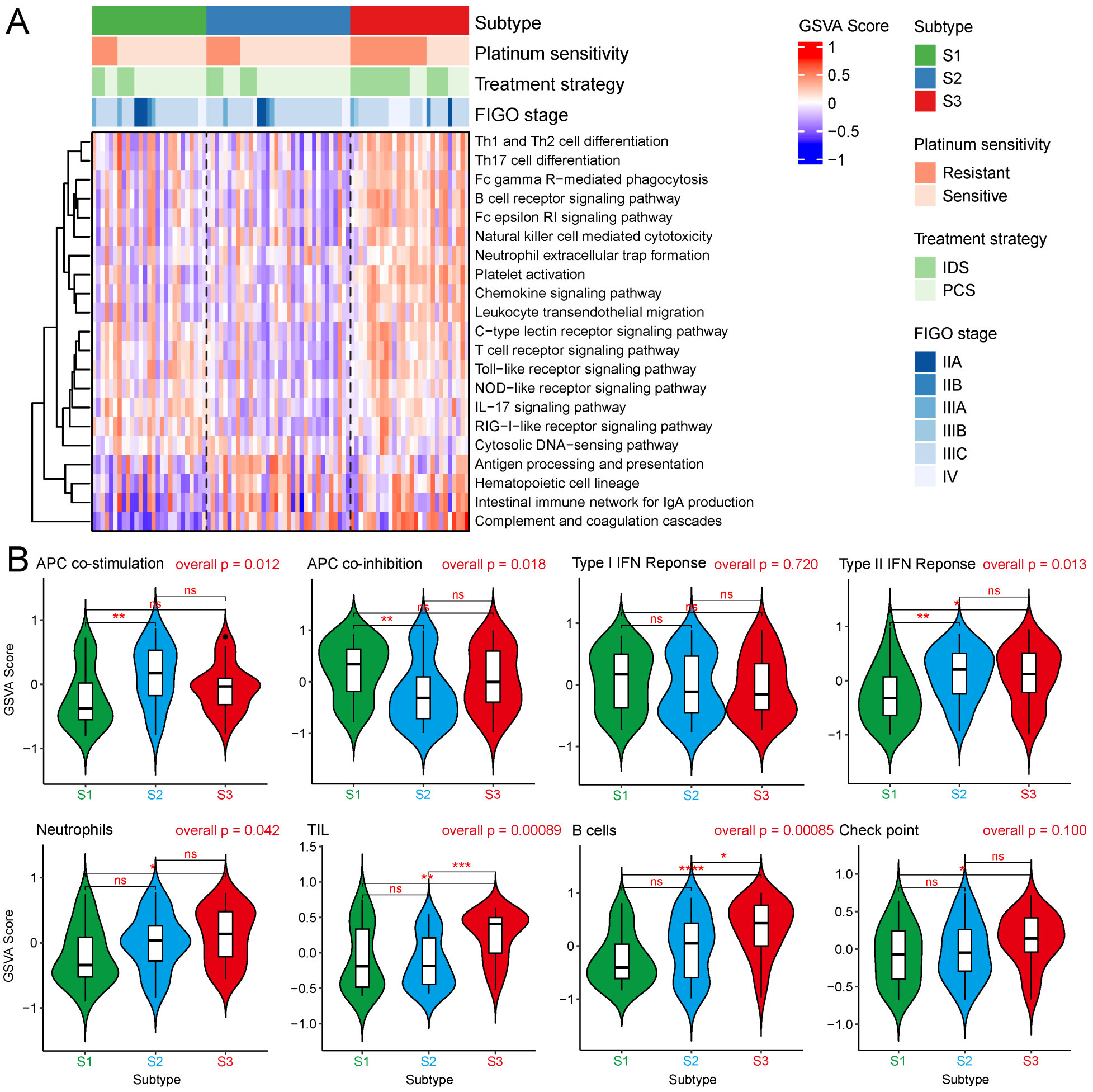

Fig. S7. Immune status of different molecular subtypes. (A) Immune pathway activation for each sample (Supplement to Figure 5A). **(B)** Enrichment of antigen-presenting cells, interferons, neutrophils, tumor-infiltrating lymphocytes, B cells, and checkpoint-related proteins in different subtypes (Supplement to Figure 5B). FIGO, International Federation of Gynecology and Obstetrics; IDS, interval debulking surgery; PCS, primary cytoreductive surgery; APC, antigen-presenting cells; IFN, interferon; TIL, tumor-infiltrating lymphocytes. The Kruskal-Wallis H test was used for comparisons among three subtypes, with the Mann-Whitney U test for subsequent pairwise comparisons. ns, no significant difference. * $p < 0.05$. ** $p < 0.01$. *** $p < 0.001$. **** $p < 0.0001$.

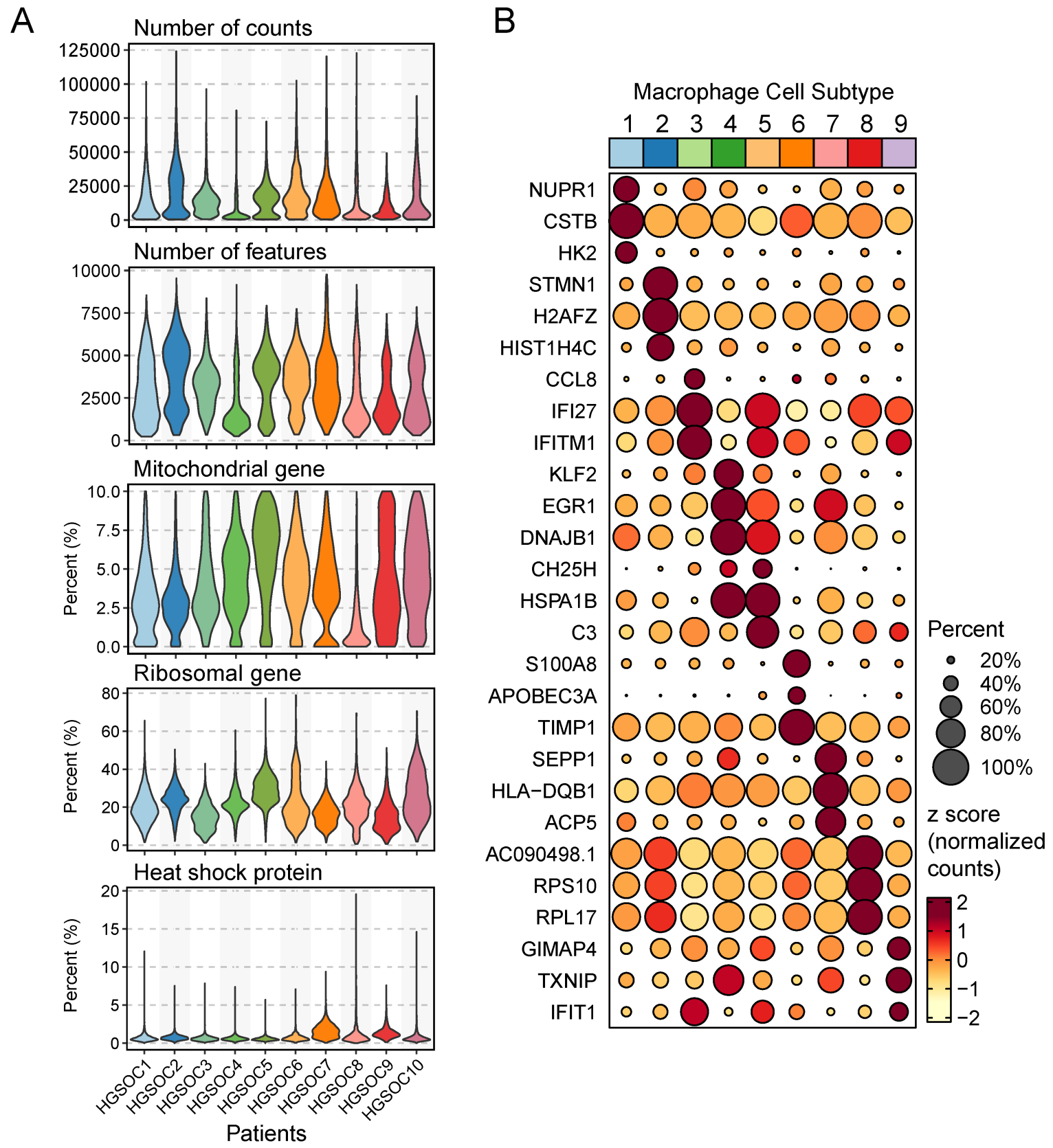

Fig. S8. Quality control and macrophage clustering for the high-grade serous ovarian cancer single-cell dataset. (Supplement to Figure 6.) **(A)** Quality control metrics. **(B)** Highly expressed genes for each macrophage subpopulation.
