# Supplementary material for: Multi‐Omics Profiling of High‐Grade Serous Ovarian Cancer Reveals an Inflammation‐Related Lipid Metabolism Subtype Associated With Platinum Resistance
Source: Adv Sci (Weinh). 2026 Jul 17:e76482. Online ahead of print. doi: 10.1002/advs.76482 (PMC13377746; doi:10.1002/advs.76482)
Supplement: Supplementary file 1 — Supporting File 1: advs76482‐sup‐0001‐SuppMat.pdf. [file ADVS-9999-e76482-s001.pdf]

Supporting Information for

**Multi-Omics Profiling of High-Grade Serous Ovarian Cancer Reveals an Inflammation-Related Lipid Metabolism Subtype Associated with Platinum Resistance**

Yuxi Zhao[#], Junyi Li[#], Bo Zheng[#], Wanshan Liu[#], Yaru Wang, Shufeng Wang, Ying Cui, Huiqin Guo, Hongxia Wang[*], Ting Xiao[*], Kun Qian[*], Jing Zuo[*]

**Table of contents**

Supplementary figures (1-8)

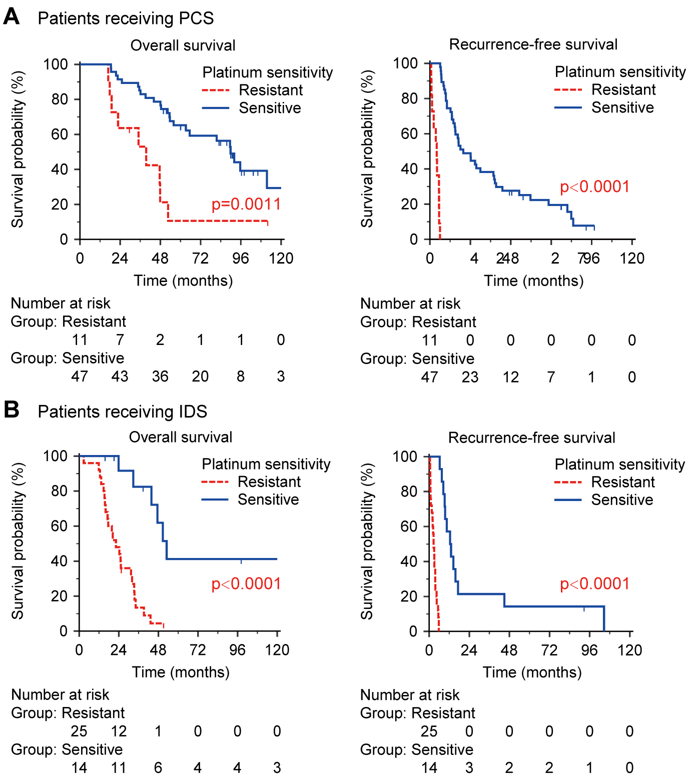


**Fig. S1. Patient prognosis based on primary cytoreductive surgery (PCS) or interval debulking surgery (IDS).** (Supplement to Figure 1.) **(A)** Overall survival (OS, left) and recurrence-free survival (RFS, right) for patients who underwent PCS. **(B)** OS (left) and RFS (right) for patients who underwent IDS. PCS, primary cytoreductive surgery; IDS, interval debulking surgery.

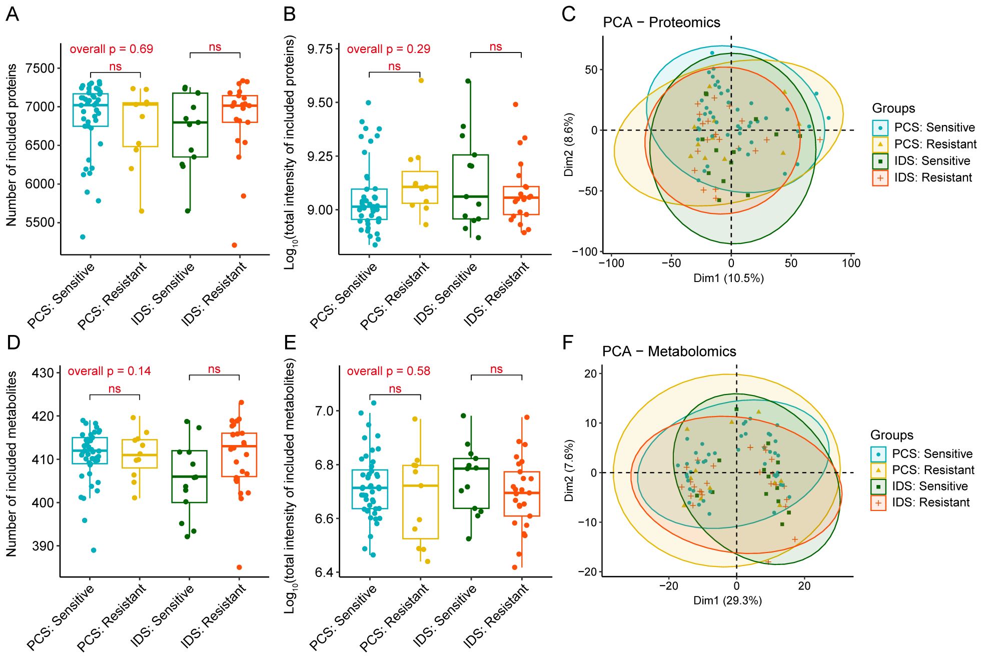


**Fig. S2. Overview of proteomic and metabolomic data across different treatment strategies and platinum sensitivities. (A)** Number of proteins included in the analysis. **(B)** Total intensity of included proteins. **(C)** Distribution of proteomic data classified by group, based on principal component analysis. **(D)** Number of metabolites included in the analysis. **(E)** Total intensity of included metabolites. **(F)** Distribution of metabolomics data classified by group, based on principal component analysis. PCS, primary cytoreductive surgery; IDS, interval debulking surgery. PCA, principal component analysis.

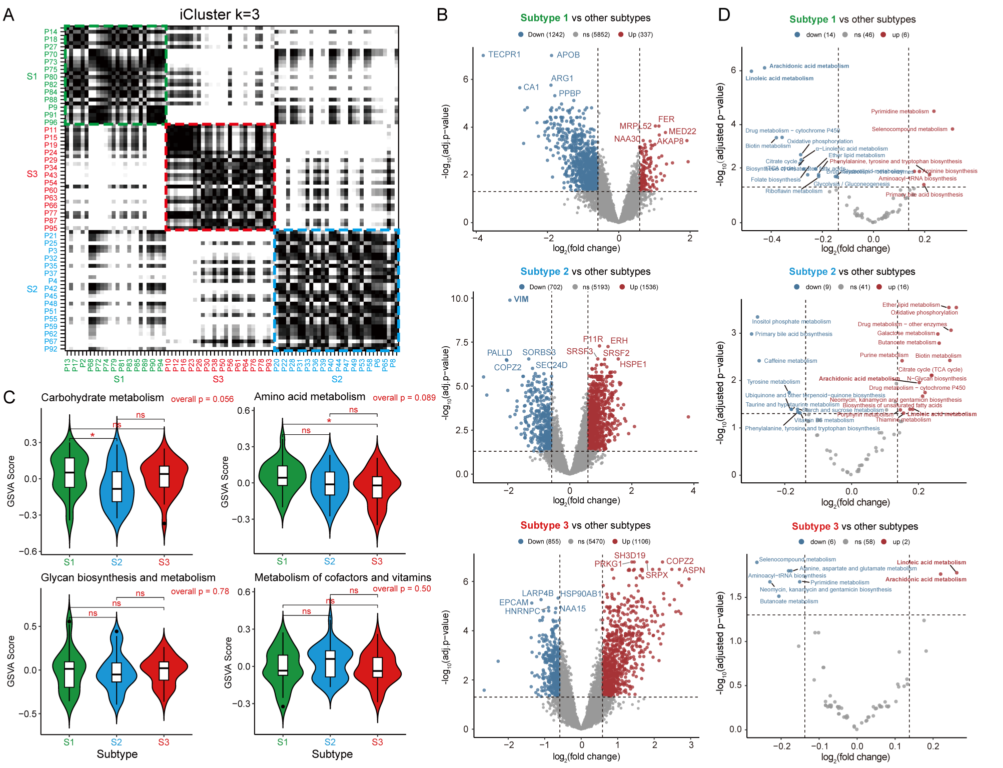


**Fig. S3. Metabolic characteristics in different molecular subtypes of ovarian cancer patients. (A)** Correlation heatmap showing patient clustering based on the iCluster algorithm (Supplement to Figure 2A). **(B)** Proteins significantly differentially expressed among patients with the three subtypes (adjusted $p < 0.05$, fold change > 1.5) (Supplement to Figure 2A). **(C)** Activation status of major metabolic types across different molecular subtypes (Supplement to Figure 3). **(D)** Metabolic pathway differences between each subtype and the remaining subtypes (Supplement to Figure 3). The Kruskal-Wallis H test was used for comparisons among three subtypes, with the Mann-Whitney U test for subsequent pairwise comparisons. ns, no significant difference. * $p < 0.05$.

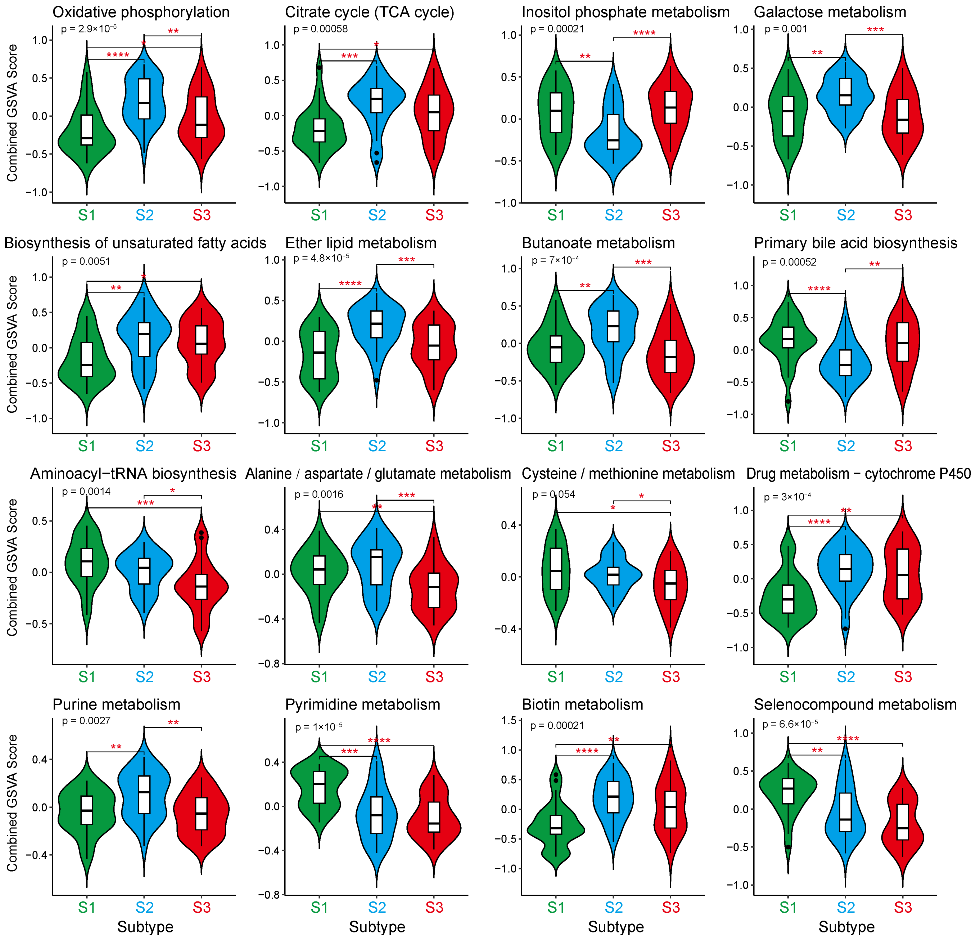


**Fig. S4. Abundance of selected metabolic pathways in different molecular subtypes.** (Supplement to Figure 3.) The Kruskal-Wallis H test was used for comparisons among three subtypes, with the Mann-Whitney U test for subsequent pairwise comparisons. * $p < 0.05$. ** $p < 0.01$. *** $p < 0.001$. **** $p < 0.0001$.

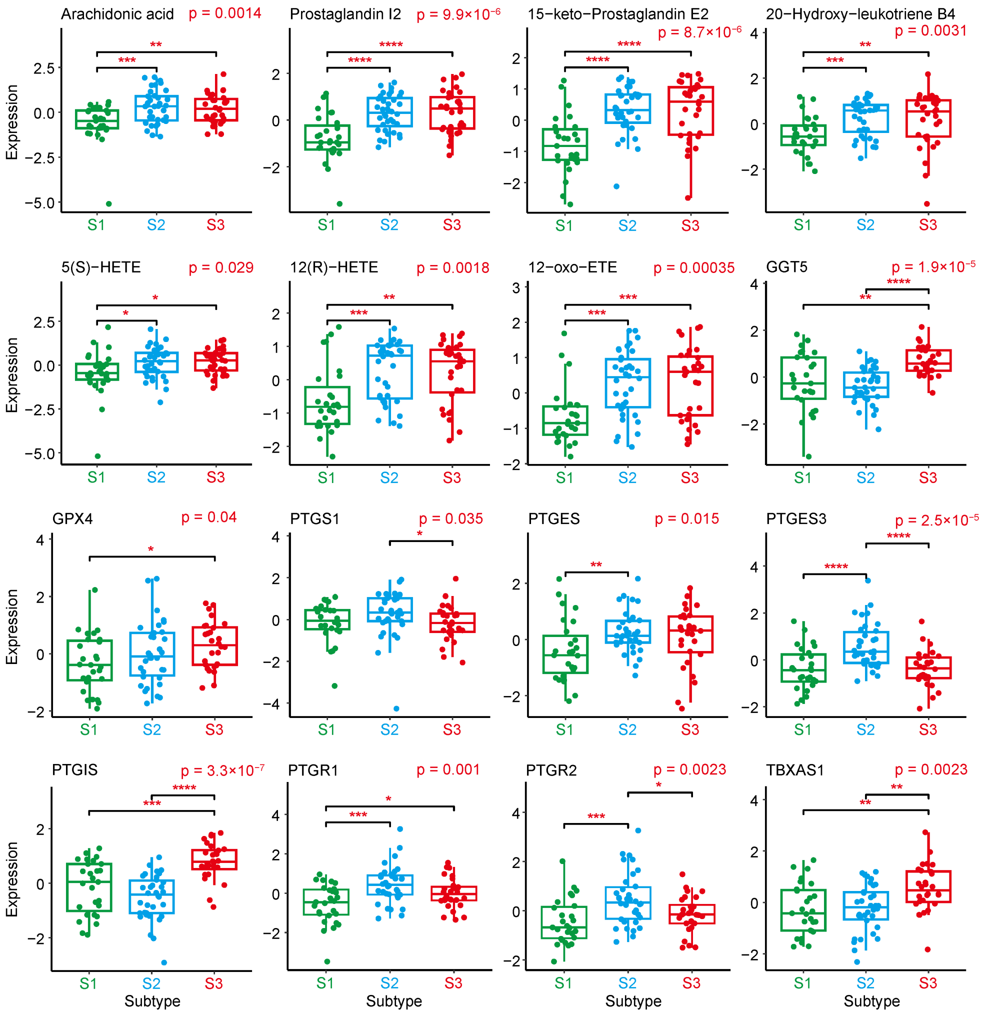


**Fig. S5. Expression or abundance of arachidonic acid metabolism-related metabolites and key proteins in different subtypes.** (Supplement to Figure 4C.) The Kruskal-Wallis H test was used for comparisons among three subtypes, with the Mann-Whitney U test for subsequent pairwise comparisons. * $p < 0.05$. ** $p < 0.01$. *** $p < 0.001$. **** $p < 0.0001$.

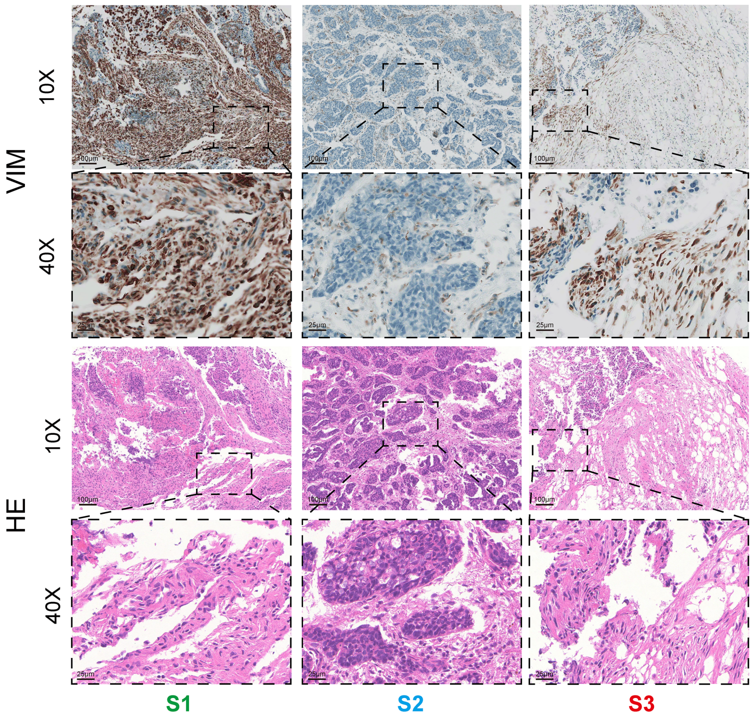


**Fig. S6. Representative staining images for immunohistochemical assessment of VIM expression levels.** (Supplement to Figure 4F.) HE, hematoxylin-eosin staining. Scale bars: 100 μm (10×) and 25 μm (40×).

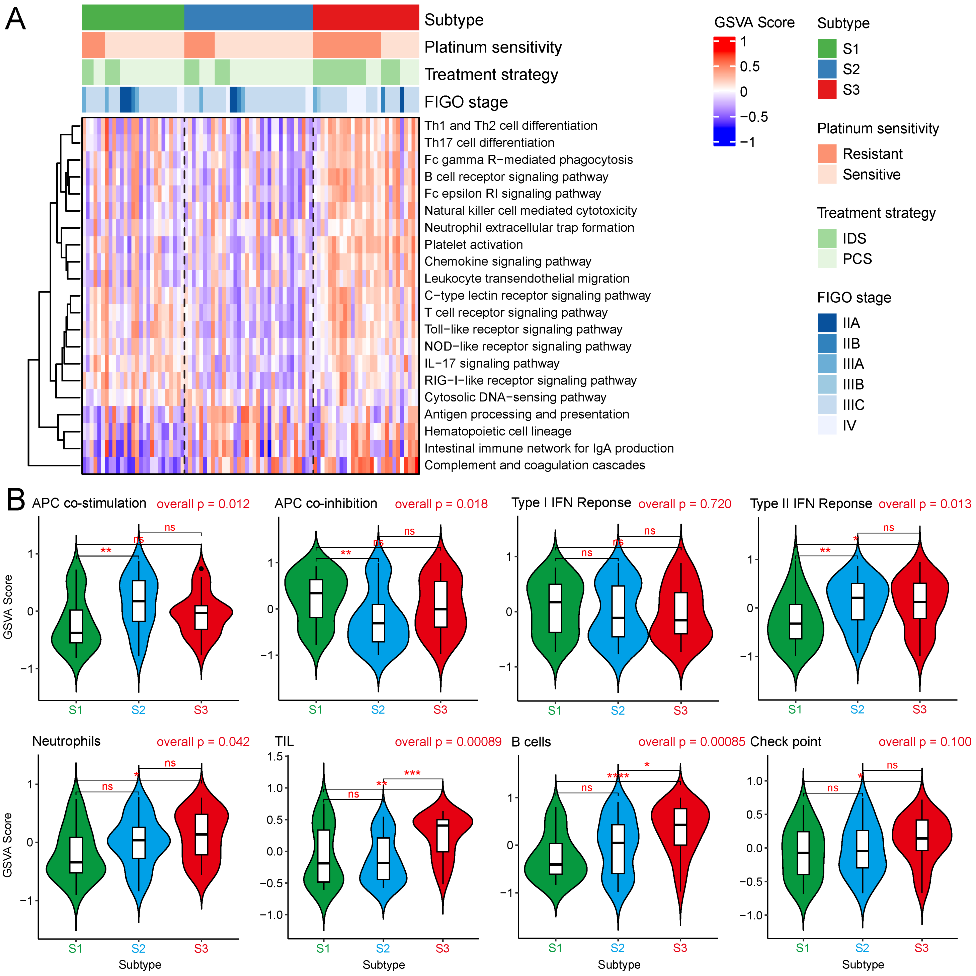


**Fig. S7. Immune status of different molecular subtypes. (A)** Immune pathway activation for each sample (Supplement to Figure 5A). **(B)** Enrichment of antigen-presenting cells, interferons, neutrophils, tumor-infiltrating lymphocytes, B cells, and checkpoint-related proteins in different subtypes (Supplement to Figure 5B). FIGO, International Federation of Gynecology and Obstetrics; IDS, interval debulking surgery; PCS, primary cytoreductive surgery; APC, antigen-presenting cells; IFN, interferon; TIL, tumor-infiltrating lymphocytes. The Kruskal-Wallis H test was used for comparisons among three subtypes, with the Mann-Whitney U test for subsequent pairwise comparisons. ns, no significant difference. * $p < 0.05$. ** $p < 0.01$. *** $p < 0.001$. **** $p < 0.0001$.

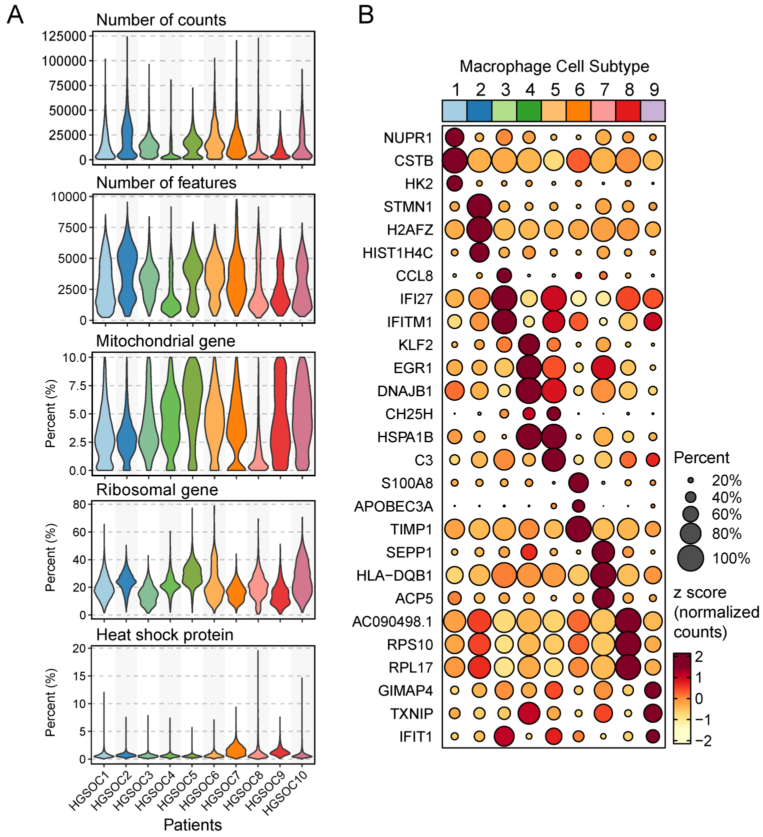


**Fig. S8. Quality control and macrophage clustering for the high-grade serous ovarian cancer single-cell dataset.** (Supplement to Figure 6.) **(A)** Quality control metrics. **(B)** Highly expressed genes for each macrophage subpopulation.
